# Study on plasma exosome miRNA sequencing and analysis in rats under hemorrhagic stress

**DOI:** 10.3389/fmed.2026.1798252

**Published:** 2026-04-16

**Authors:** Junxin Zhou, Jiaxiang Gu, Shui Xiong, Lang Jiang, Xiang Gao, Gaorong Deng

**Affiliations:** 1 Department of Orthopaedics, Affiliated Rehabilitation Hospital of Nanchang University, Nanchang, Jiangxi, China; 2 Scoliosis Center of the Fourth Affiliated Hospital of Nanchang University, Nanchang, Jiangxi, China; 3 Department of Orthopaedics, Jiujiang Municipal Hospital of Traditional Chinese Medicine, Nanchang, Jiangxi, China

**Keywords:** bioinformatics, exosomes, hemorrhagic shock, miRNA sequencing, rat model

## Abstract

This study explored the impact of hemorrhagic stress on the expression of plasma exosome miRNAs in rats using high-throughput miRNA sequencing technology. The results revealed that hemorrhagic stress significantly altered the expression profiles of multiple miRNAs, particularly the upregulation of miRNA-193b-3p, which is associated with vascular repair, and the downregulation of miRNA-485-5p, which may be related to impaired cell repair and antioxidant responses. Further target gene prediction and pathway analysis suggested that the differentially expressed miRNAs play potential roles in key physiological processes such as immune response, cell repair, and angiogenesis, especially in the enrichment of the TNF and MAPK signaling pathways. These findings suggest that these miRNAs may play important roles in the recovery process following hemorrhage by regulating these pathways. These results provide new evidence for the biological functions of miRNAs in hemorrhagic stress and offer potential biomarkers and therapeutic targets for early diagnosis and intervention. Although this study provides new insights into the role of exosome miRNAs in hemorrhagic stress, there are certain limitations, such as the use of only a rat model in the experiment. The broader applicability of the results requires further validation through other animal models or clinical studies.

## 1 Introduction

Hemorrhagic Shock (HS) is a circulatory failure caused by acute massive blood loss, commonly seen in clinical situations such as trauma, surgery, and internal bleeding (1). It not only directly affects the body’s hemodynamics and metabolic functions but also triggers widespread immune responses and cellular damage, potentially leading to multi-organ failure (2). Despite ongoing advancements in medicine, fluid resuscitation, blood transfusion, and pharmacological treatments can somewhat alleviate the symptoms of hemorrhagic shock, but it remains a major cause of patient mortality (3). The treatment of hemorrhagic shock is challenging due to its diverse clinical manifestations and the complexity of treatment (4). Therefore, an in-depth study of the molecular mechanisms of hemorrhagic shock, especially the identification of new biomarkers, is of great significance for improving early diagnostic accuracy and providing a basis for therapeutic interventions (5).

The pathological mechanism of hemorrhagic shock is highly complex, often involving multiple physiological responses such as blood volume reduction, hypotension, and hypoxia (6). When blood loss reaches a certain level, blood pressure drops sharply, leading to insufficient perfusion of vital organs (such as the brain, heart, kidneys, etc.), which in turn affects their normal functions (7). At the same time, tissues and cells begin to undergo metabolic disturbances due to hypoxia and inadequate nutrient supply, resulting in impaired cell function and cell death (8). Another key feature of hemorrhagic shock is the alteration of the immune system. After blood loss, the body’s immune response undergoes significant changes, including the activation of immune cells, excessive release of inflammatory factors, and the occurrence of immune suppression (9). These responses not only may exacerbate the inflammatory response but also weaken the body’s immune defense, increasing the risk of secondary infections, which can even progress to sepsis, one of the major causes of death in hemorrhagic shock patients (10). Clinically, the assessment of hemorrhagic shock mainly relies on monitoring clinical parameters such as blood pressure, heart rate, and blood oxygen saturation. However, these indicators often cannot accurately reflect the severity of the condition (11). Existing biomarkers, such as lactate and white blood cell count, although helpful in monitoring hemorrhagic shock, still have limitations in terms of sensitivity and specificity (12). Therefore, accurately identifying hemorrhagic shock, especially in the early stages, is crucial for optimizing treatment strategies and improving patient prognosis. In this context, exosomes, as an emerging biomarker carrier, have gradually become an important direction in the study of hemorrhagic shock (13).

Exosomes are membrane vesicles released from the multivesicular bodies (MVBs), which contain multiple membrane structures, typically ranging from 30 to 150 nanometers in diameter (14). They facilitate intercellular communication by carrying and transferring biomolecules such as miRNA, mRNA, and proteins, thereby regulating the functions of target cells (15). In recent years, exosomes have gradually become a key area of research in pathological processes such as intercellular signal transduction, immune regulation, and diseases like cancer and neurodegenerative disorders (16). Exosomes not only serve as carriers for intercellular communication but also show great potential in disease mechanism research, diagnosis, and therapy, particularly in studies of cancer, immune-related diseases, and neurodegenerative diseases, where their role is particularly significant (17, 18).

Exosomes have highly heterogeneous biological functions, with molecular components carried by exosomes differing across various cell types (19). For example, exosomes secreted by tumor cells are enriched in miRNAs and proteins related to tumor progression, while exosomes secreted by immune cells may contain immune-regulatory factors. These exosomes not only participate in the regulation of local immune responses but can also be transmitted through body fluids such as blood, influencing the function of distant tissues (20). In immune responses, the miRNAs carried by exosomes play a crucial role. By transferring miRNAs produced by immune cells, they affect the activity, proliferation, and differentiation of other immune cells, thus regulating the intensity and direction of the immune response (21). For example, miRNA-155 and miRNA-21 play important roles in immune responses, respectively regulating T cell differentiation, B cell activation, immune suppression, and inflammatory responses (22). The expression changes of these miRNAs not only affect immune system function but also contribute to the onset and progression of various immune-related diseases (23).

In the study of hemorrhagic shock, exosomes have also shown important biological functions (24). Exosomes, as membrane vesicles secreted by cells, are widely present in body fluids such as blood, urine, and saliva, and they can carry various biomolecules, particularly miRNAs, which regulate key physiological processes such as immune response, inflammation, and cell repair (25). In hemorrhagic shock, exosomes regulate the body’s immune and inflammatory responses through the miRNAs and other molecules they carry, thus influencing the pathological process (26). Therefore, studying the miRNAs within exosomes, especially their changes in hemorrhagic shock, may provide new biomarkers and potential therapeutic targets for early diagnosis and treatment (27). By deeply exploring the role of exosomes in hemorrhagic shock, it is expected to provide new strategies for clinical treatment and improve patient prognosis (28).

Exosomes play important roles in many physiological and pathological processes, particularly in key physiological processes such as intercellular communication, immune response regulation, cell repair, and angiogenesis (29). As mediators of intercellular signaling, exosomes can carry various biomolecules, including miRNAs, mRNAs, and proteins, to regulate the functions of target cells (30). Therefore, exosomes and the miRNAs they carry have significant potential for application in early disease diagnosis, treatment monitoring, and clinical intervention (31). Especially in the context of hemorrhagic shock, the miRNAs carried by exosomes may help the body recover and repair by regulating processes such as immune response, vascular repair, and tissue regeneration (32). Factors such as the sharp decline in blood flow and cell hypoxia caused by hemorrhagic shock typically lead to the generation of significant inflammatory responses and affect the functions of immune cells and blood vessels (33). The miRNAs in exosomes may play a critical role in the recovery process following hemorrhage by regulating these physiological processes (34).

Previous studies have shown that the regulation of the immune system is significantly affected under hemorrhagic shock, with the expression of certain miRNAs changing notably (35). For example, miRNA-21 has been shown to have an immune-regulatory role during hemorrhagic shock, enhancing anti-inflammatory responses and promoting cell repair by regulating the function of immune cells (36). miRNA-155 plays a crucial role in the inflammatory response, regulating the activation of immune cells and the release of cytokines (37). Furthermore, exosome miRNAs may also participate in the angiogenesis process, promoting the repair of damaged blood vessels and the generation of new vessels, which is vital for recovery after hemorrhage (38). After hemorrhagic shock, the restoration of vascular function and the generation of new blood vessels are critical physiological processes. Exosome miRNAs may play an essential role in this process by regulating pathways such as the VEGF signaling pathway (39). To better understand the role of exosome miRNAs in hemorrhagic shock, this study aims to analyze the miRNA expression profiles in plasma exosomes from rats under hemorrhagic shock using high-throughput miRNA sequencing technology, and to explore their potential molecular mechanisms (40). We will use ultracentrifugation to extract plasma exosomes from rats and employ transmission electron microscopy (TEM) to morphologically characterize the exosomes, confirming their size and concentration. Additionally, bioinformatics methods will be applied to analyze the differential expression of miRNAs and their potential target genes and functional pathways, revealing the molecular functions of exosome miRNAs in hemorrhagic shock. This will provide new theoretical evidence and practical guidance for early diagnosis and treatment (41).

## 2 Research and methods

### 2.1 Animal models and experimental groups

In this study, adult male Sprague–Dawley (SD) rats (8–10 weeks old, weighing 250–300 grams) were used. All rats were acclimated under standard breeding conditions (22–24 °C, 50 ± 10% humidity, 12-h light/dark cycle). To minimize confounding factors, all surgical procedures and sample collections were consistently performed between 9:00 a.m. and 2:00 p.m. Sample size was determined by power analysis based on a preliminary study (*α* = 0.05, Power = 0.8), ensuring sufficient statistical sensitivity to detect a 2-fold change in miRNA expression. Animals were randomly assigned to groups using a random number table.

To establish a fixed-volume hemorrhagic shock model, rats underwent jugular vein cannulation under anesthesia. Following a 30-min stabilization period to ensure baseline Mean Arterial Pressure (MAP) and heart rate stability, hemorrhage was initiated. The hemorrhagic stress was induced by withdrawing 35% of the total blood volume over 15 min to simulate acute traumatic blood loss. The shock state was maintained for a duration of 2 h, during which MAP was continuously monitored to ensure it remained within the range of 40 ± 5 mmHg. After the 2-h shock period, immediate resuscitation was performed by re-infusing the withdrawn blood combined with an equal volume of normal saline over 30 min. Successful model construction was verified by physiological indicators, including a significant drop in MAP during shock and an elevation of blood lactate levels (>4.0 mmol/L) measured via a blood gas analyzer prior to resuscitation.

To establish a hemorrhagic stress rat model, we employed a standard blood loss method. Rats underwent blood loss via jugular vein cannulation, Following cannulation, a 30-min stabilization period was allowed to ensure that the Mean Arterial Pressure (MAP) and heart rate returned to stable baseline levels before initiating hemorrhage, with a blood volume loss of 30–40% of body weight to simulate the pathological state of traumatic hemorrhagic stress. After blood loss, immediate resuscitation with fluids (including saline and other resuscitative solutions) was performed to restore blood volume and ensure physiological stability. The experimental groups are as follows:

Control group (Group A)

This group served as a sham-operated control. To eliminate the potential interference of the surgical procedure on hemodynamic status, rats in Group A underwent the exact same anesthesia and jugular vein cannulation as the experimental group, but without blood withdrawal or fluid resuscitation. This group of rats was not subjected to any treatment and served as the normal control group, with physiological changes observed without hemorrhagic stress. The specific groupings are as follows:

- DSXJ_A-1
- DSXJ_A-2
- DSXJ_A-3

Experimental group (Group B, hemorrhagic stress group)

This group of rats underwent standard blood loss treatment to simulate traumatic hemorrhagic stress. The specific groupings are as follows:

- DSXJ_B-1
- DSXJ_B-2
- DSXJ_B-3

### 2.2 Exosome extraction and purification

In this study, exosomes were extracted using ultracentrifugation. Regarding the sampling timeline, plasma samples were collected immediately (0 h) following the completion of fluid resuscitation to capture the acute regulatory response of miRNAs to hemorrhagic stress and reperfusion. To ensure the rigor of the study, all sample labeling and downstream processing, including exosome isolation and sequencing, were performed by researchers blinded to the experimental groupings. Approximately 5 mL of whole blood was collected into EDTA tubes and processed via differential centrifugation at 4 °C: 2,000×*g* for 30 min to remove cells, and 10,000×*g* for 30 min to remove debris. Exosomes were then isolated by ultracentrifugation at 100,000×*g* for 2 h, washed with PBS, and finally resuspended in 200 μL PBS for characterization and RNA extraction. Then, the plasma was further centrifuged at 10,000×*g* for 30 min at 4 °C to remove larger vesicles and cell debris. The supernatant was then transferred to a new centrifuge tube and ultracentrifuged at 100,000×*g* for 2 h at 4 °C. The supernatant was discarded, and the pellet was washed with PBS and ultracentrifuged again at 100,000 × g for 2 h at 4 °C. Finally, the exosome sample was resuspended in a fixed volume of 200 μL PBS and stored at −80 °C.

- Ultracentrifugation for exosome extraction: The filtered liquid was transferred to a new centrifuge tube and centrifuged at 100,000×*g* for 90 min at 4 °C using an ultracentrifuge (Hitachi CP100MX). After centrifugation, the supernatant was carefully removed, and the exosome pellet was resuspended in 1 × PBS solution. To further purify the exosomes, the pellet was subjected to a second round of centrifugation at 100,000×*g* for 90 min. Finally, the exosome sample was resuspended in 200 μL PBS and stored at −80 °C.

### 2.3 Exosome characterization and particle size analysis

The extracted exosomes were characterized using Scanning Electron Microscopy (SEM), Transmission Electron Microscopy (TEM), and particle size analysis to confirm their morphology, membrane integrity, and physical properties. While TEM is a standard for observing cross-sectional lipid bilayers, we incorporated high-resolution SEM due to its superiority in providing intuitive three-dimensional (3D) surface topology and a broader field of view for population assessment.

- Scanning Electron Microscopy (SEM) Observation: To better capture the characteristic 3D structure of the vesicles, a 10 μL aliquot of the sample was fixed in 2.5% glutaraldehyde, dehydrated through a graded series of ethanol, and critically point dried. The samples were then gold-coated and observed under a field-emission SEM. SEM provides a distinct advantage in visualizing the typical “cup-shaped” morphology—a hallmark of exosomes resulting from dehydration—which is more clearly defined than in 2D TEM projections. Furthermore, SEM allows for a more comprehensive evaluation of sample purity and the absence of large-scale aggregation.
- Transmission Electron Microscopy (TEM) Observation: For morphological characterization of the internal and bilayer structures, a 10 μL aliquot of the 200 μL resuspended exosome sample was dropped onto a copper grid and left to settle for 1 min. The excess liquid was removed using filter paper, and the sample was stained with 2% phosphotungstic acid for 1 min before being observed under a TEM.
- Particle Size Analysis (NanoFCM): The particle size and concentration of the exosomes were measured using the NanoFCM particle size analyzer. A 10 μL sample was taken and appropriately diluted according to the instrument’s operating instructions. After calibration with standard samples, the instrument analyzed the sample and provided information on the particle size distribution and concentration. The particle size distribution should fall within the 30-150 nm range, confirming the typical size of the exosomes.

### 2.4 miRNA extraction and high-throughput sequencing

Total RNA extraction from the remaining 180 μL of collected exosome suspension was performed using the RNeasy Plus Micro Kit (Qiagen). The concentration and purity of the extracted RNA samples were quantified using a NanoDrop spectrophotometer. For each sample, the total RNA yield was ensured to be at least 200 ng (with a concentration > 10 ng/μL), providing sufficient high-quality material for subsequent analyses. The extracted miRNA was used for library construction using the Illumina TruSeq Small RNA Library Prep Kit, and high-throughput sequencing was performed on the Illumina HiSeq 2,500 platform.

- miRNA Library Construction and Sequencing: The extracted miRNA was used for library construction with the Illumina TruSeq Small RNA Library Prep Kit, following the instructions provided by the kit. The constructed miRNA library was subjected to high-throughput sequencing on the Illumina HiSeq 2,500 platform. The sequencing data was stored in FASTQ format, and raw data underwent quality control and trimming to remove low-quality sequences, ensuring data reliability.

### 2.5 miRNA data analysis and differential expression analysis

All sequencing data were aligned using the Cufflinks tool from the Tuxedo software suite. miRNA sequencing data were first aligned with the miRBase (version 22) database using Bowtie2. After alignment, differential expression analysis was performed using EdgeR or DESeq2 to identify miRNAs that showed significant differential expression between the hemorrhagic stress group and the control group. To further validate the function of differentially expressed miRNAs, miRNA target gene prediction was carried out using tools such as miRanda and TargetScan.

- Differential miRNA Screening: miRNA expression data between the hemorrhagic stress group and the control group were compared. miRNAs with statistical significance (*p* < 0.05) and significant fold changes were selected for further analysis. The results of differential miRNA screening provide the basis for subsequent biological function analysis.
- Bioinformatics Analysis: GO (Gene Ontology) and KEGG (Kyoto Encyclopedia of Genes and Genomes) pathway analyses were performed to investigate the target genes of differentially expressed miRNAs and the biological processes and pathways they participate in. Functional enrichment analysis helps reveal the potential roles of these miRNAs in immune regulation, cell repair, angiogenesis, and other processes.

### 2.6 Statistical analysis

All data are presented as mean ± standard deviation (Mean ± SD) and were analyzed using SPSS software. Differential miRNA expression was compared using *t*-tests, with *p* < 0.05 considered statistically significant. Functional enrichment analysis of differentially expressed miRNAs was conducted using R language and the ClusterProfiler software, and the results were presented in the form of heatmaps, volcano plots, etc.

### 2.7 Ethical statement

All animal experiments in this study were strictly conducted in accordance with the “Regulations on the Administration of Laboratory Animals” and relevant provisions of the Animal Ethics Committee of our institution. In all procedures, the welfare of the animals was fully considered, and efforts were made to minimize pain and discomfort during the experiments.

## 3 Results

### 3.1 Exosome extraction and characterization

Exosomes were extracted from DSXJ_A and DSXJ_B samples, and their morphology and particle size distribution were characterized using Scanning Electron Microscopy (SEM) and Dynamic Light Scattering (DLS).

**Figure 1:**
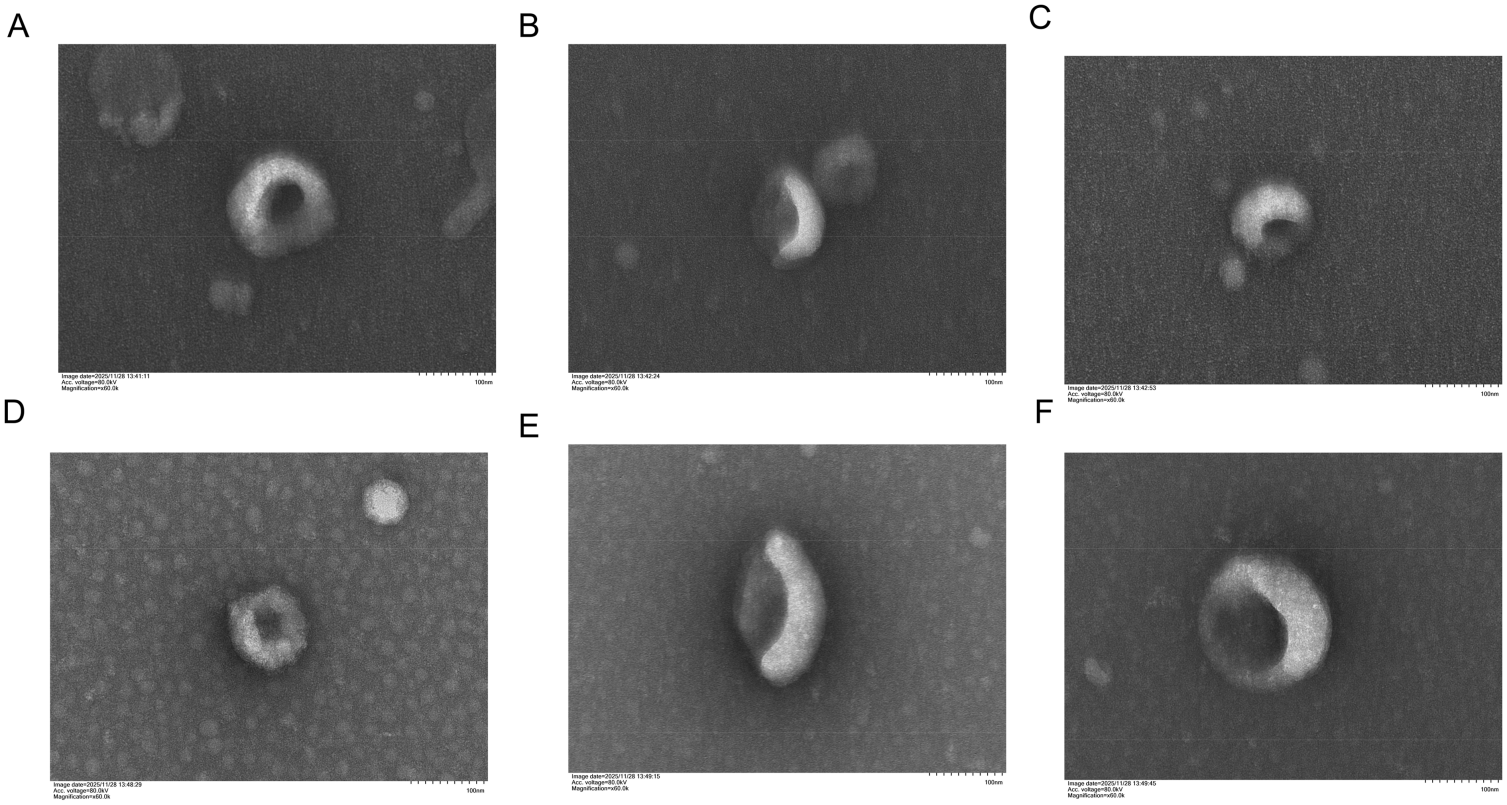
Typical morphology of exosomes from DSXJ_A group and DSXJ_B group within the 100 nm range. **(A–C)** Show the typical morphology of exosomes from the DSXJ_A group, and **(D–F)** show the typical morphology of exosomes from the DSXJ_B group. A: DSXJ_A.100 nm.1, B: DSXJ_A.100 nm.2, C: DSXJ_A.100 nm.3, D: DSXJ_B.100 nm.1, E: DSXJ_B.100 nm.2, F: DSXJ_B.100 nm.3. All images display the typical bilayer membrane structure, with the exosome particle size range concentrated around 100 nm.

First, the SEM images at a 100 nm scale (Figure 1) demonstrated the typical bilayer structure of the exosomes. The exosome particles from DSXJ_A (Figures 1A–C) and DSXJ_B (Figures 1D–F) both exhibited consistent spherical morphology, further confirming that the morphological characteristics of the exosomes from both groups were consistent and clear at this scale. Next, Supplementary Figure S1 displays the exosome morphology at different particle size scales. At the 500 nm scale (Supplementary Figures S1A,B), both DSXJ_A and DSXJ_B exosomes exhibited the typical bilayer structure, with the particles clearly showing spherical shapes. At the 1 μm scale (Supplementary Figures S1C,D), the exosomes maintained a similar bilayer structure, and the particle boundaries were clearly visible, indicating that the exosome morphology of DSXJ_A and DSXJ_B was well-defined and met expectations.

**Figure 2:**
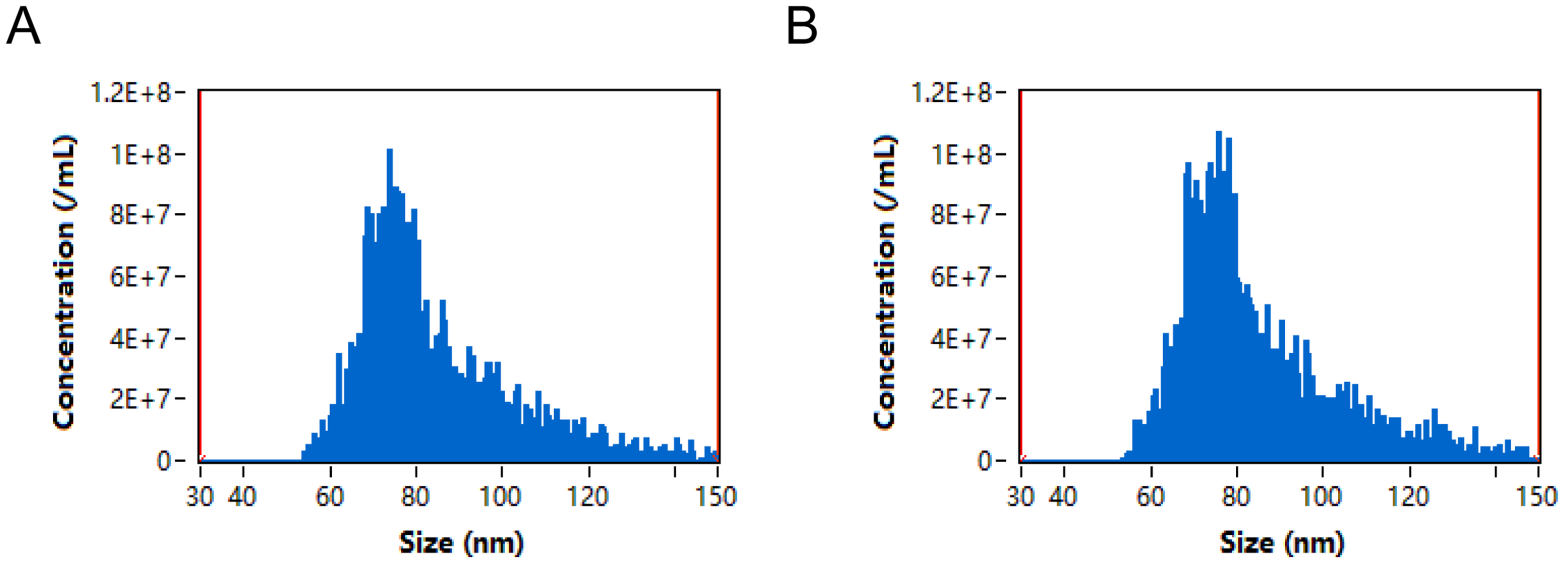
Particle size distribution of exosomes from the DSXJ_A and DSXJ_B groups. Figure shows the particle size distribution of exosomes from the DSXJ_A and DSXJ_B groups. **(A)** Particle size distribution of exosomes from the DSXJ_A group, primarily concentrated in the 80–90 nm range. **(B)** Particle size distribution of exosomes from the DSXJ_B group, also mainly concentrated in the 80–90 nm range. The particle size distributions of the exosomes from both groups show high similarity, exhibiting a uniform distribution characteristic.

To further analyze the exosome particle size distribution, we performed Dynamic Light Scattering (DLS), and the results are shown in Figure 2. The particle size distribution of DSXJ_A (Figure 2A) showed that the main peak was concentrated in the 80–90 nm range, which is typical for exosome size. The particle size distribution of DSXJ_B (Figure 2B) was similar to DSXJ_A, with the main peak also in the 80–90 nm range. The particle size distributions of both samples were highly concentrated, suggesting that the exosome populations had high homogeneity.

### 3.2 miRNA high-throughput sequencing and quality control

In this study, we used high-throughput sequencing technology to sequence miRNA from multiple samples, combined with strict quality control steps to ensure the reliability of the data and the accuracy of subsequent analyses.

#### 3.2.1 Data sequencing quality analysis

**Figure 3:**
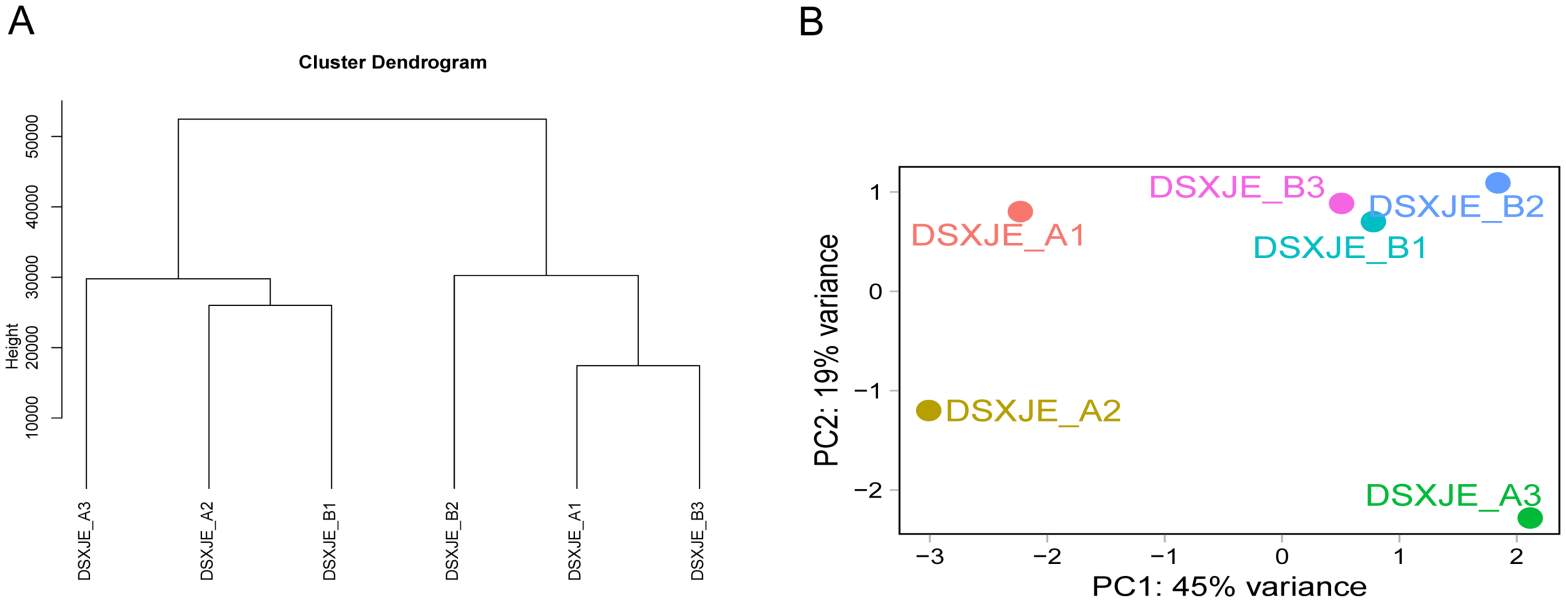
Euclidean distance clustering and PCA analysis of the samples. **(A)** The Euclidean distance clustering dendrogram of the samples, which shows the similarity between samples. The grouping relationships of the samples are obtained through the clustering algorithm. **(B)** The PCA distribution plot based on the DESeq2 analysis results, showing the distribution of the samples along the first two principal components (PC1 and PC2). PC1 explains 45% of the variance, and PC2 explains 19% of the variance, highlighting the distribution of the samples and potential group differences.

All samples in this study underwent high-quality sequencing to ensure the reliability of the data and the accuracy of subsequent analyses. Figure 3 shows the Euclidean distance clustering and Principal Component Analysis (PCA) results, reflecting the quality and similarity between the samples. To evaluate the reliability of the sequencing data and the biological similarity among samples, hierarchical clustering analysis was performed (Figure 3A). The results indicated a general trend of separation between the experimental groups. Although samples A1 and B1 exhibited some degree of cross-clustering in the dendrogram—potentially reflecting the individual biological heterogeneity of plasma exosomal miRNA expression profiles or phased variations in physiological response to hemorrhagic stress—the overall technical consistency remained high. This is further supported by the quality control metrics in Supplementary Table S1, where all samples achieved Q30 scores greater than 94%. Moreover, the spatial distribution in the PCA plot (Figure 3B) demonstrated a distinct separation between Group A and Group B. These findings confirm that the dataset possesses sufficient reliability and stability for subsequent differential expression analysis.

The PCA analysis in Figure 3B further illustrates a distinct separation between Group A (DSXJE_A1, A2, A3) and Group B (DSXJE_B1, B2, B3) within the principal component space. This spatial distribution indicates substantial differences in the miRNA expression profiles between the control and hemorrhagic stress groups, rather than reflecting technical quality variances. While the samples from Group B clustered tightly together, the broader distribution of Group A samples reflects the baseline biological heterogeneity among individual rats. These PCA results, combined with the sequencing metrics, confirm that the data characteristics are robust enough to distinguish the convergent biological effects of hemorrhagic stress from individual variations in the control group.

Further analysis of the individual sample data is shown in Supplementary Table S1, which presents the sequencing quality metrics for each sample. For example, the DSXJE_A1 sample had 14,036 raw reads, a Q20 value of 91.21%, a Q30 value of 84.57%, and a GC content of 59%, indicating good sequencing data quality. After data cleaning, the Q20 value of DSXJE_A1 increased significantly to 98.78%, the Q30 value improved to 96.10%, and the GC content remained at 52% (Supplementary Table S1). This quality improvement demonstrates the importance of data cleaning in enhancing data quality. These data show that, whether raw or cleaned, all samples met high-quality standards, and the increase in Q20 and Q30 values confirmed the importance of data cleaning in improving data quality.

#### 3.2.2 Distribution of RNA categories

Supplementary Table S3 presents the alignment results of each sample with the Rfam database, analyzing the distribution of different types of small RNAs. From the data in Supplementary Table S2, it can be seen that there are some differences in the distribution of small RNAs across samples. For instance, in the DSXJE_A1 sample, the number of Cis-reg RNAs was 9,756, rRNAs were 145,034, and the total RNA count was 1,081,021; while in the DSXJE_B1 sample, the number of Cis-reg RNAs was 9,618, rRNAs were 145,808, and the total RNA count was 901,725 (Supplementary Table S2). Despite the large number of rRNAs in both groups (constituting a significant proportion of total RNA), the A group had generally higher numbers of lncRNAs and Cis-reg RNAs, while the B group had a relatively higher proportion of rRNAs. This suggests that the A group is rich in lncRNAs and Cis-reg RNAs, which may have specific miRNA characteristics.

Additionally, Supplementary Table S2 presents the read counts for small RNA lengths across different samples. The length distribution of small RNAs exhibited some variation between samples (Supplementary Table S2).

**Figure 4:**
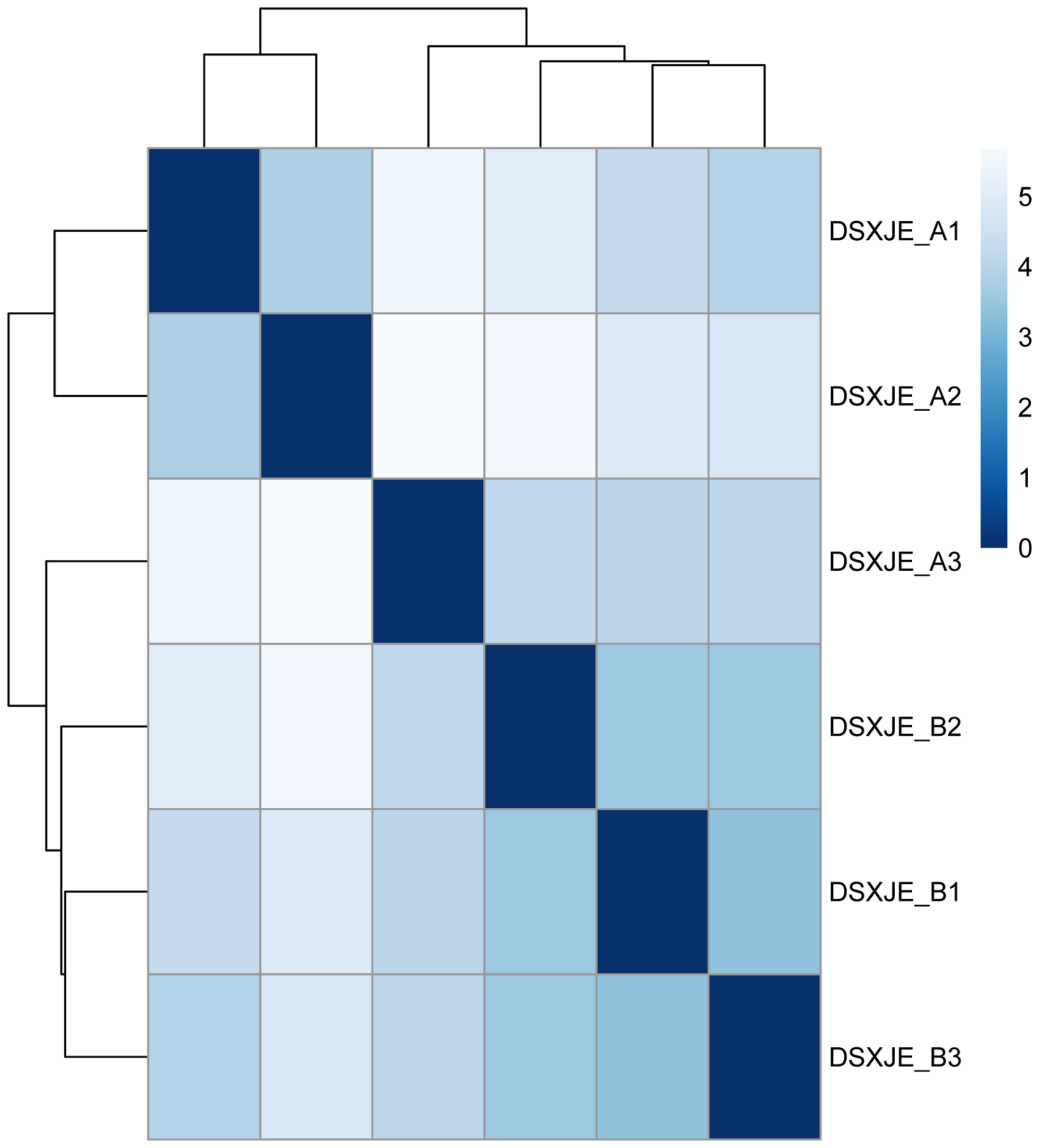
Sample distance analysis based on DESeq2.

**Figure 5:**
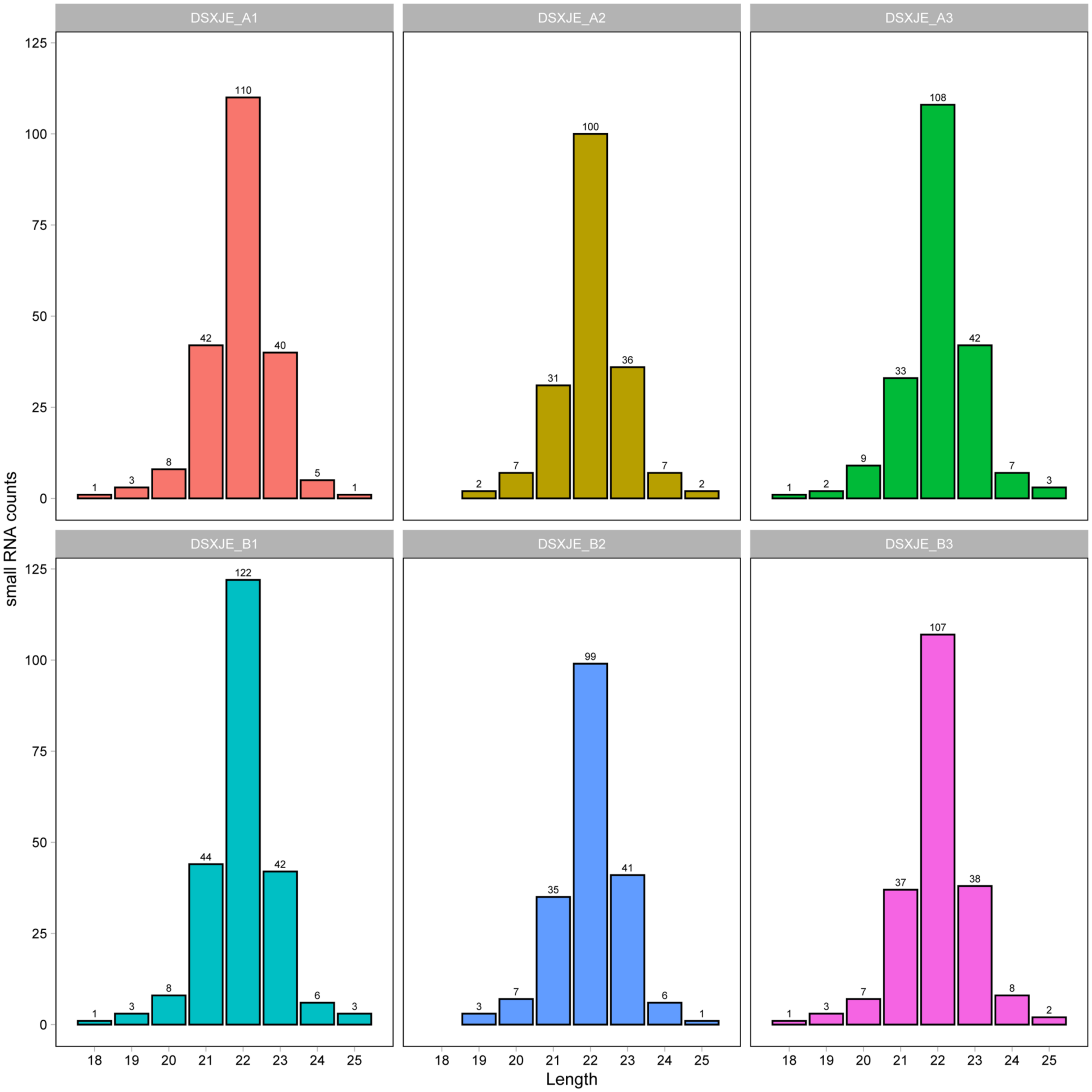
Distribution of small RNA lengths. Figure shows the length distribution of small RNAs in different samples. Each subplot displays the distribution of small RNA counts for various lengths (ranging from 18 to 25 nucleotides) in a single sample. The *X*-axis represents the length of the small RNAs (in nucleotides), while the *Y*-axis represents the count of small RNAs (the number of small RNAs). Different colors are used for each sample to help distinguish their distribution characteristics. From the figure, it can be observed that most small RNAs are enriched within a specific length range, particularly in the 21–23 nucleotide range, with the distribution peaks varying between samples.

Furthermore, the heatmap in Figure 4 compares the distances between the samples, further illustrating the differences in RNA category distributions. The sample distance analysis in the heatmap aligns with the results above, with A group samples showing greater consistency in their classification, while the B group displays more dispersion, indicating significant differences in RNA category distribution between the samples (Figure 4). The histogram in Figure 5 further illustrates the distribution of small RNA lengths, providing a more intuitive view of the differences in small RNA lengths between the A and B groups (Figure 5).

Figure 4 shows the heatmap of the distance matrix between samples based on DESeq2 analysis. The heatmap displays the similarity or difference between different samples, where the color intensity represents the distance between samples. Darker colors indicate greater distances between samples, while lighter colors indicate closer distances. Through hierarchical clustering, the samples are divided into several groups. The clustering results show that the similarity within groups is high, while the differences between groups are significant.

The samples include DSXJE_A1, DSXJE_A2, DSXJE_A3, DSXJE_B1, DSXJE_B2, DSXJE_B3, etc. The clustering analysis helps identify similarity patterns between the samples, providing a basis for subsequent data analysis.

Note: The color bar in the heatmap represents the relative distance between the samples, with darker blue indicating greater differences between samples and lighter blue indicating higher similarity between samples.

### 3.3 Differential miRNA screening and analysis

#### 3.3.1 Differential miRNA screening and expression analysis

Through further analysis of the miRNA expression data, we found that hemorrhagic stress significantly altered the expression profiles of multiple miRNAs in plasma exosomes. Specifically, several miRNAs with statistically significant differences were identified between Group B and Group A.

**Figure 6:**
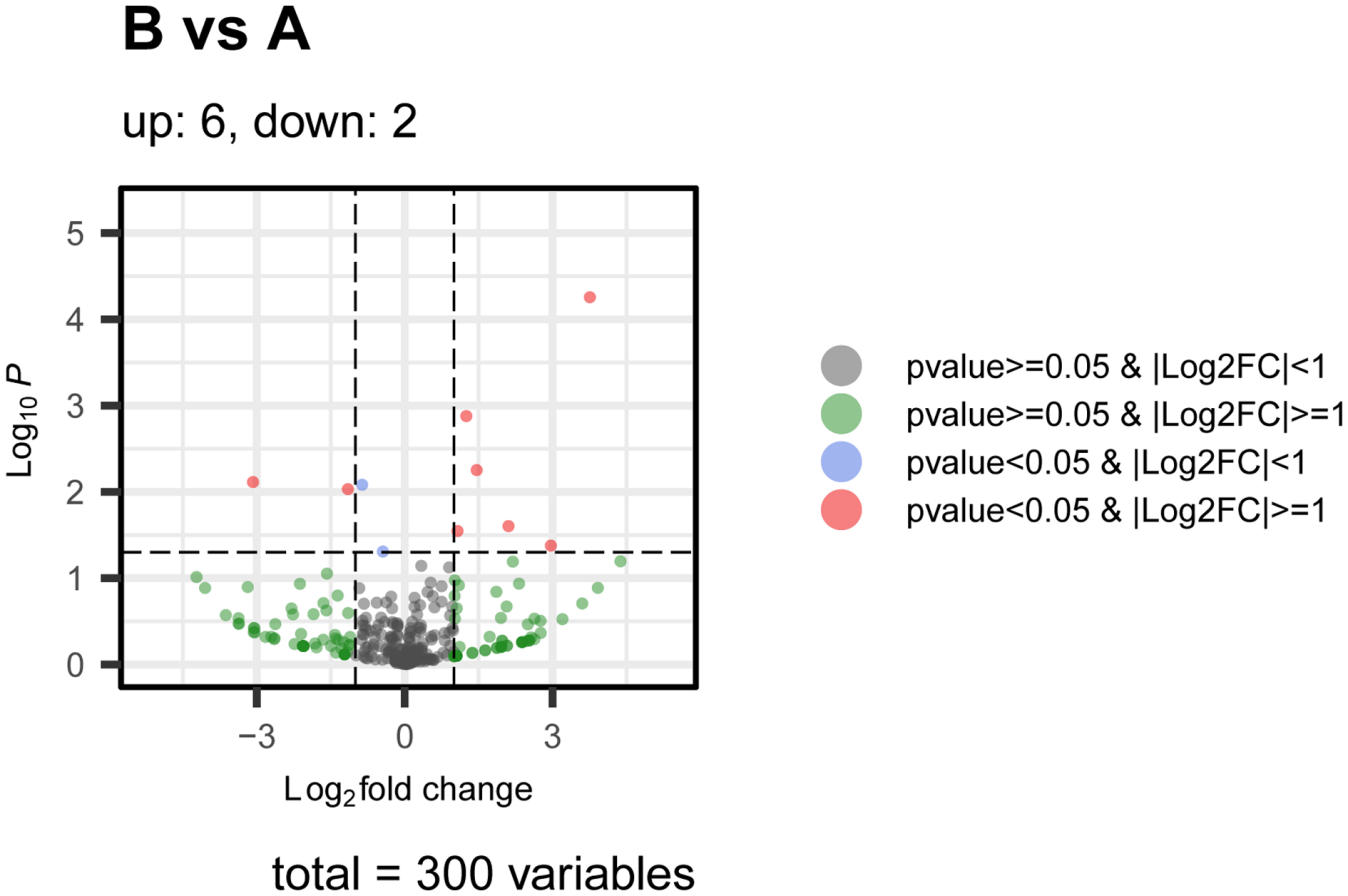
Volcano plot of miRNA between B and A. Figure shows the volcano plot of miRNA differential analysis results between group B and group A. The *X*-axis represents the log-transformed fold change (Log_2_ fold change), and the *Y*-axis represents the negative log of the *p*-value (Log_10_ *P*). Each point represents a miRNA, and the color and position of the points indicate the statistical significance and expression changes of that miRNA. Based on the thresholds of *p*-value and fold change, the points are categorized into four types: Gray points: *p*-value ≥ 0.05 and |Log2 FC| < 1, indicating miRNAs with no significant difference or small expression changes. Blue points: *p*-value < 0.05 and |Log2 FC| < 1, indicating miRNAs with significant differences but small expression changes. Green points: *p*-value < 0.05 and |Log2 FC| ≥ 1, indicating miRNAs with significant differences and large downregulation in expression. Red points: *p*-value < 0.05 and |Log2 FC| ≥ 1, indicating miRNAs with significant differences and large upregulation in expression. The plot labels the number of upregulated and downregulated miRNAs, which are 6 upregulated and 2 downregulated, respectively. The distribution in the volcano plot highlights the miRNAs that show statistically significant differences and substantial expression changes between group B and group A.

First, rno-miR-193b-3p was significantly upregulated in Group B, with a Log2 fold change of 4.38 and a *p*-value of 0.006634 (Supplementary Table S5), indicating that its expression level in Group B was 22 times higher than in Group A (Figure 6). High-throughput sequencing revealed a distinct shift in the plasma exosomal miRNA profile under hemorrhagic stress. Notably, miRNA-193b-3p and miRNA-485-5p emerged as the most significantly altered transcripts, exhibiting marked upregulation (log2FC = 4.38) and downregulation (log2FC = −4.22), respectively (*p* < 0.01).

rno-miR-1b also showed significant downregulation in Group B (Log2 fold change of −3.76, *p*-value = 5.54E−06), with a decrease in its expression that could reflect enhanced cell apoptosis and tissue damage induced by hemorrhagic stress. Apoptosis plays a critical role in the recovery process after blood loss, potentially affecting tissue regeneration and repair (Figure 6).

Additionally, rno-miR-92b-5p (Log2 fold change of −4.41, *p*-value = 0.129480) and rno-miR-205-5p (Log2 fold change of 3.92, *p*-value = 0.129480) showed differences between Group B and Group A, but the differences did not reach statistical significance (Supplementary Table S5). This suggests that the expression changes of these miRNAs may be influenced by sample variation or other physiological factors, rather than being directly caused by hemorrhagic stress. Further studies on these miRNAs may uncover their potential roles in other physiological or pathological conditions.

#### 3.3.2 Differential miRNA screening and quality control

**Figure 7:**
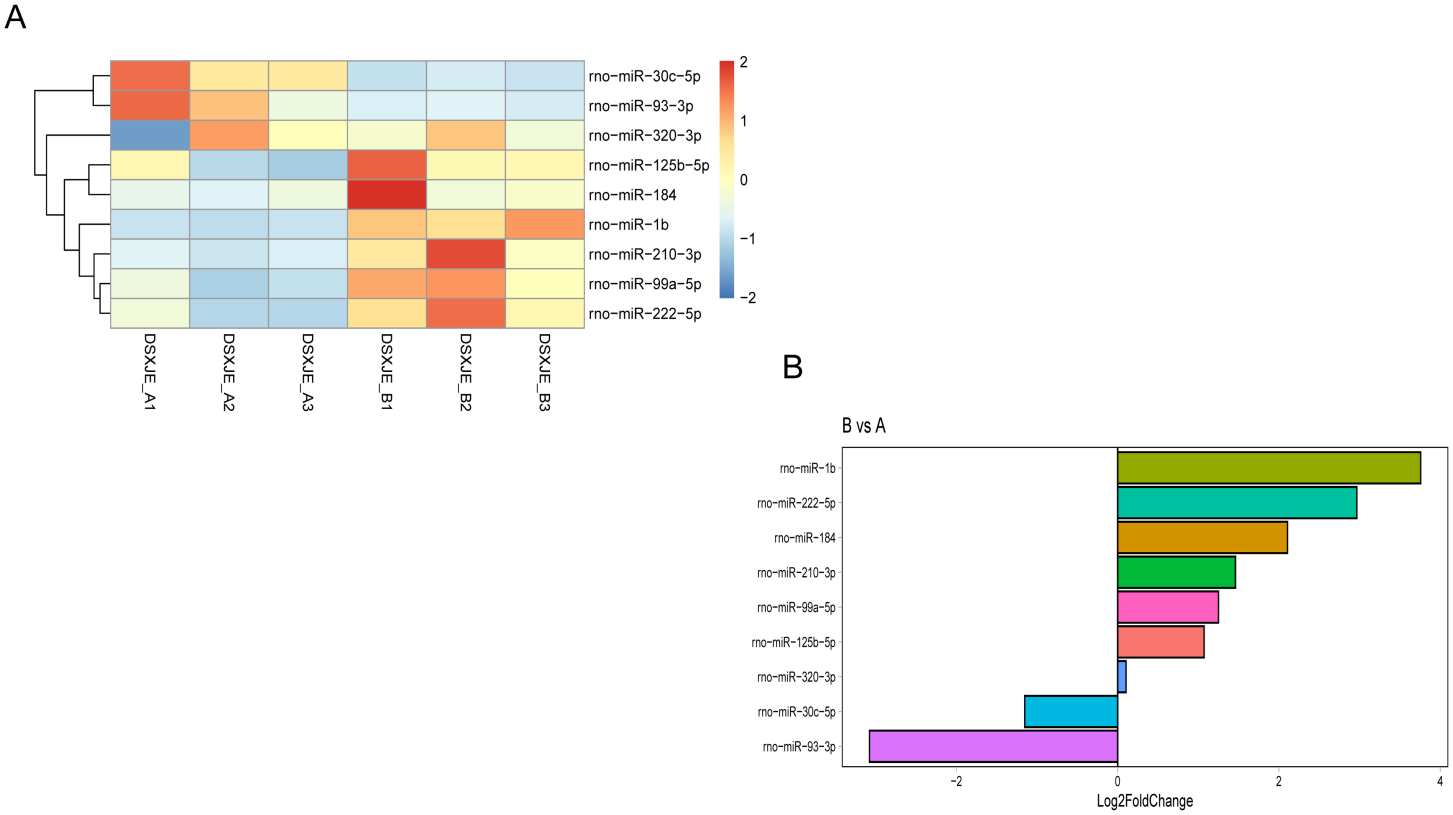
Differential miRNA expression analysis between group B and group A. Figure shows the differential miRNA expression analysis between group B and group A. The figure consists of two parts: **(A)** The heatmap displays the expression profiles of differentially expressed miRNAs between group B and group A. Each row represents a miRNA, and each column represents a sample. The color gradient from blue to red indicates changes in miRNA expression levels, with darker colors representing higher expression. Hierarchical clustering is applied to group the samples and miRNAs based on their expression patterns, allowing for observation of expression differences. **(B)** The bar chart shows the Log2 fold change (Log2 Fold Change) of differentially expressed miRNAs between group B and group A. Each bar represents a miRNA, with the color of the bar indicating the specific miRNA, and the length of the bar reflecting the expression difference of that miRNA. The chart highlights the miRNAs that are significantly upregulated or downregulated between group B and group A. Part A illustrates the expression patterns of miRNAs in group B and group A, while Part B visually displays the expression changes of these miRNAs, emphasizing the significantly upregulated and downregulated miRNAs.

In this study, we systematically screened and analyzed the miRNA expression differences between plasma exosomes from normal rats (Group A) and plasma exosomes from hemorrhagic stress rats (Group B). The consistency of the samples was validated through Principal Component Analysis (PCA) and clustering analysis. The results showed a clear separation between the A and B groups. Notably, samples within Group B exhibited high consistency and tight clustering, indicating a uniform biological response to hemorrhagic stress, whereas Group A showed higher biological variability at baseline (Figures 3B, 7A). This result ensures that the observed differential miRNA expression changes were induced by hemorrhagic stress rather than by sample handling or sequencing biases.

During the miRNA screening process, we applied a threshold of Log2 fold change (log2FoldChange) greater than 1.5 and a *p*-value less than 0.05, which helped to accurately identify miRNAs with significant differential expression between Group B and Group A (Supplementary Tables S4, S5). Using these criteria, we identified a batch of miRNAs with significantly different expression levels from the plasma exosomes of the two groups. Some of these miRNAs were significantly upregulated in Group B, while others were significantly downregulated (Figure 7B). For example, rno-miR-1b showed significant upregulation in Group B, while rno-miR-93-3p showed significant downregulation in Group B. These differential miRNAs provide reliable evidence for further understanding the potential roles of miRNAs during hemorrhagic stress.

Additionally, rno-miR-92b-5p (Log2 fold change of −4.41, *p*-value = 0.129480) and rno-miR-205-5p (Log2 fold change of 3.92, *p*-value = 0.129480) showed differences between Group B and Group A, but the differences did not reach statistical significance (Supplementary Table S5). This suggests that the expression changes of these miRNAs may be influenced by sample variation or other physiological factors, rather than being directly caused by hemorrhagic stress. Further studies on these miRNAs may uncover their potential roles in other physiological or pathological conditions.

### 3.4 miRNA target gene prediction and pathway analysis

In this study, we performed target gene prediction and pathway analysis for the differentially expressed miRNAs between Group B and Group A. By integrating miRNA target gene validation data, Gene Ontology (GO) analysis, and KEGG pathway analysis, we thoroughly investigated the potential roles of these differentially expressed miRNAs in various biological processes and signaling pathways. This in-depth analysis further clarified their functions in both physiological and pathological states.

Through these analyses, we gained insights into how these miRNAs may influence key biological processes such as immune response, cell repair, apoptosis, and angiogenesis, which are crucial in the context of hemorrhagic stress and recovery. These findings could help to deepen our understanding of the role of miRNAs in various disease states and inform future therapeutic strategies targeting miRNAs.

#### 3.4.1 miRNA target gene function and annotation

First, based on the differentially expressed miRNAs, we performed target gene prediction and functional validation. Supplementary Table S3 provides relevant information about these miRNA target genes, including miRNAs (such as rno-miR-30c-5p, rno-miR-125b-5p, etc.) and their target genes (such as Camk2d, Tp53, and Ccn2). To further clarify the biological functions of these target genes, we conducted Gene Ontology (GO) analysis.

According to the results in Supplementary Table S6, miRNA target genes were significantly enriched in several biological processes. Notably, in the GO entry “fluid transport” (GO:0042044), the differentially expressed target genes exhibited strong enrichment (GeneRatio = 0.017751, *p*.adjust = 0.048113), suggesting that miRNAs may play a key role in fluid balance and metabolism. Additionally, miRNA target genes were enriched in GO entries related to immune response and cellular adaptation, providing important evidence for the potential functions of miRNAs in inflammatory responses, cell proliferation, and cardiovascular health (Supplementary Table S4).

**Figure 8:**
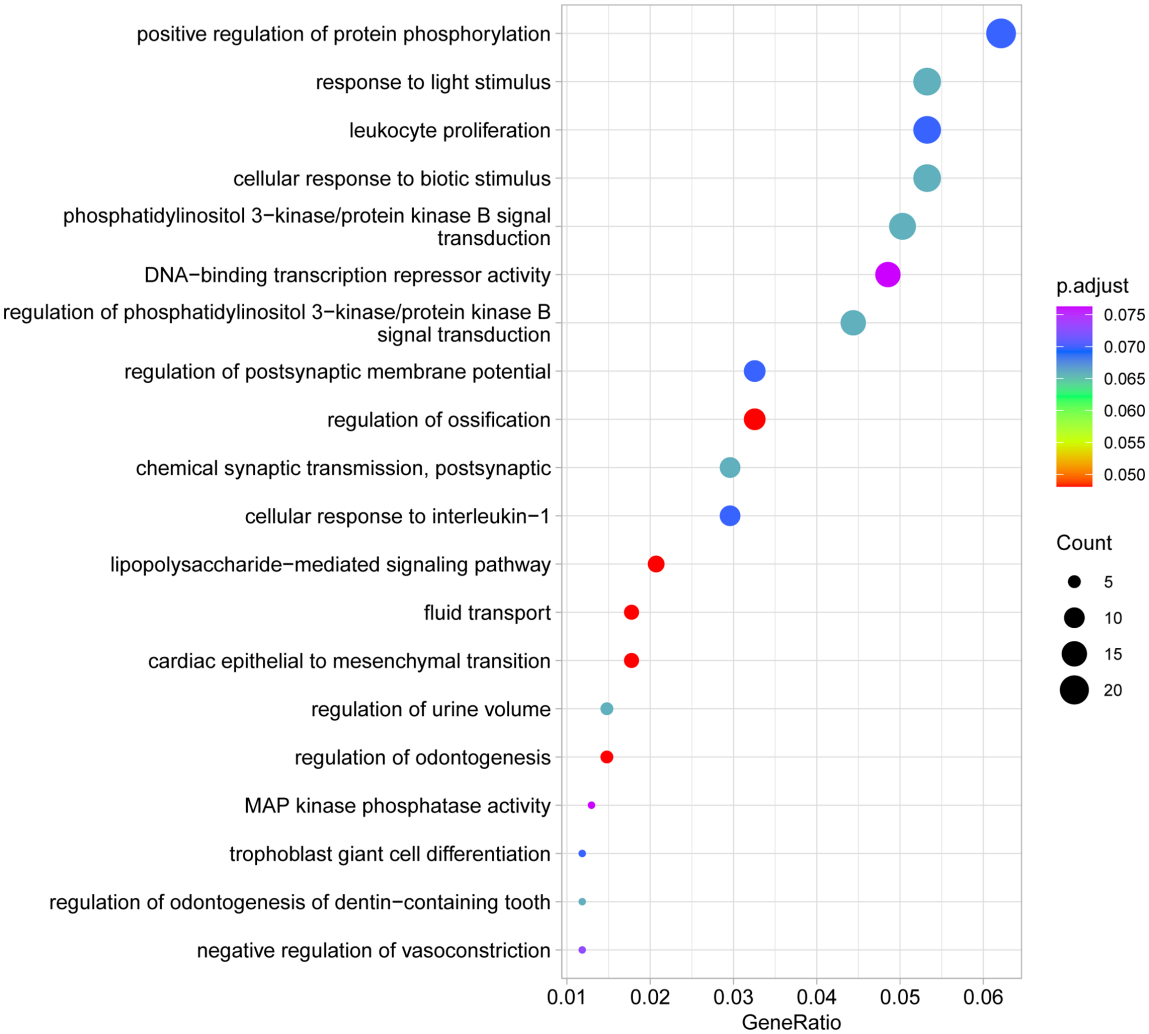
Top 20 GO terms of target genes validated for differentially expressed miRNAs between group B and group A. The figure shows the top 20 functional terms obtained through GO analysis, sorted by Gene Ratio. The size of each circle represents the number of genes involved in that functional term (Count), and the color of the circle indicates the adjusted *p*-value (*p*.adjust), with the color gradient from red (smaller *p*-value) to blue (larger *p*-value). The functional terms displayed include biological processes related to cellular responses, transcriptional regulation, signal transduction, and more, such as “positive regulation of protein phosphorylation,” “response to light stimulus,” and “leukocyte proliferation”. This figure presents the major functional categories of DE miRNA target genes from the GO analysis, reflecting their potential roles in various biological processes.

Figure 8 shows the enrichment of miRNA target genes in the top 20 GO terms. By comparing the GeneRatio and *p*.adjust values, we found that miRNA target genes are not only involved in fluid transport but also enriched in processes such as “positive regulation of protein phosphorylation” (GO:0042325) and “cellular response to light stimulus” (GO:0060610), indicating their important roles in cellular adaptation, signal transduction, and protein regulation. For example, the enrichment of the process “positive regulation of protein phosphorylation” (GeneRatio = 0.027, *p*.adjust = 0.013) in miRNA regulation suggests that miRNAs might play a critical role in cell signaling and gene expression regulation by influencing the expression of related genes.

#### 3.4.2 KEGG pathway analysis of miRNA target genes

**Figure 9:**
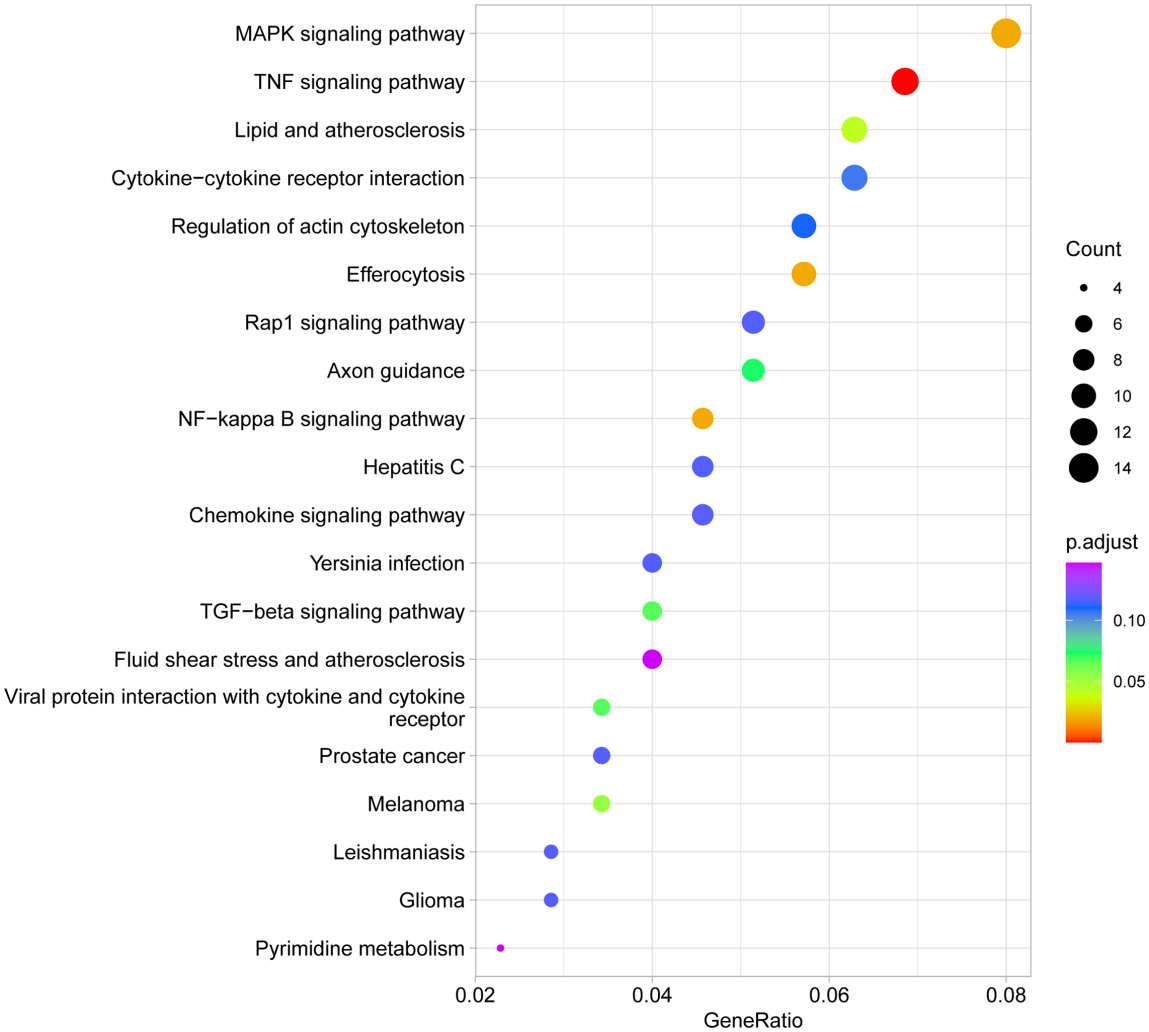
Top 20 KEGG pathways of target genes validated for differentially expressed miRNAs between group B and group A. This figure illustrates the major signaling pathways enriched in the KEGG analysis of DE miRNA target genes, reflecting their potential impact on cellular functions and diseases. KEGG pathway analysis of DE miRNA target genes. The figure displays the top 20 significantly enriched pathways in the KEGG analysis for DE miRNA target genes. The size of each circle represents the number of genes involved in that pathway (Count), and the color of the circle indicates the adjusted *p*-value (*p*.adjust), with the color gradient from red (smaller *p*-value) to blue (larger *p*-value). The figure highlights several important biological pathways, including the “MAPK signaling pathway,” “TNF signaling pathway,” and “lipid metabolism and atherosclerosis,” indicating the potential roles of these pathways in DE miRNA regulation.

To elucidate the roles of miRNA target genes in biological processes, KEGG pathway analysis was performed. As shown in Supplementary Table S7 and Figure 9, differentially expressed miRNA target genes were significantly enriched in several key signaling pathways, most notably the TNF signaling pathway (rno04668; GeneRatio = 0.0685, *p*.adjust < 0.001) and MAPK signaling pathway (rno04010; GeneRatio = 0.0800, *p*.adjust = 0.019). Additionally, the lipid and atherosclerosis pathway (rno05417) and NF-kappa B signaling pathway showed significant enrichment. These findings suggest that miRNAs exert systemic control over inflammatory responses, cell proliferation, and metabolic homeostasis by modulating these core signaling networks during hemorrhagic stress.

#### 3.4.3 miRNA target genes and disease relevance

The functional analysis of miRNA target genes also revealed their potential roles in various diseases. Through GO and KEGG analyses, we discovered that miRNAs may regulate immune responses, cell proliferation, and metabolic processes by modulating pathways such as the TNF signaling pathway and MAPK signaling pathway. For example, the enrichment in the TNF signaling pathway suggests that miRNAs may play a critical role in cancer, infectious diseases, and autoimmune diseases. The enrichment in the MAPK signaling pathway indicates that miRNAs could be involved in the initiation and progression of tumors by regulating genes associated with the cell cycle.

Additionally, by modulating genes in the lipid and atherosclerosis pathway, miRNAs may impact cardiovascular diseases, diabetes, and other metabolic disorders. By targeting genes such as Scarb1 and Irf3, miRNAs may play a significant role in metabolism, inflammation, and cardiovascular health. The potential involvement of miRNAs in these diseases warrants further research to better understand their mechanisms and therapeutic applications in various disease contexts (Table 1).

**Table 1:** Verified target genes of differentially expressed miRNAs between B and A groups after filtering.

Database	mature_mirna_id	target_symbol	target_entrez	Experiment	support_type	Type
mirtarbase	rno-miR-30c-5p	Camk2d	24,246	Luciferase reporter assay//qRT-PCR//Western blot	Functional MTI	Validated
mirtarbase	rno-miR-30c-5p	Tp53	24,842	Luciferase reporter assay//Western blot	Functional MTI	Validated
mirtarbase	rno-miR-30c-5p	Ccn2	64,032	Luciferase reporter assay//Western blot	Functional MTI	Validated
mirtarbase	rno-miR-99a-5p	Bmpr2	140,590	Luciferase reporter assay	Functional MTI	Validated
mirtarbase	rno-miR-125b-5p	Sema4d	306,790	Immunohistochemistry//Luciferase reporter assay//Microarray//Western blot	Functional MTI	Validated
mirtarbase	rno-miR-125b-5p	Cyp24a1	25,279	Luciferase reporter assay//Northern blot//qRT-PCR	Functional MTI	Validated
mirtarbase	rno-miR-210-3p	Fech	361,338	Luciferase reporter assay//Microarray//qRT-PCR//Western blot	Functional MTI	Validated

The filtering criteria are clearly listed, ensuring that the selected miRNA target gene data have been reliably validated and are suitable for further analysis.

This table provides a brief summary of the verified miRNA target genes, focusing on the interactions between the differentially expressed miRNAs in Group B and Group A and their target genes.

The selected miRNAs belong to the rno-miR family, which is commonly used in rat model studies, and their target genes are involved in key biological processes such as cell cycle regulation and apoptosis.

The interactions validated by experimental methods such as luciferase reporter assays provide reliability to these data, making them suitable for further pathway analysis.

### 3.5 miRNA target gene prediction and experimental validation analysis

**Figure 10:**
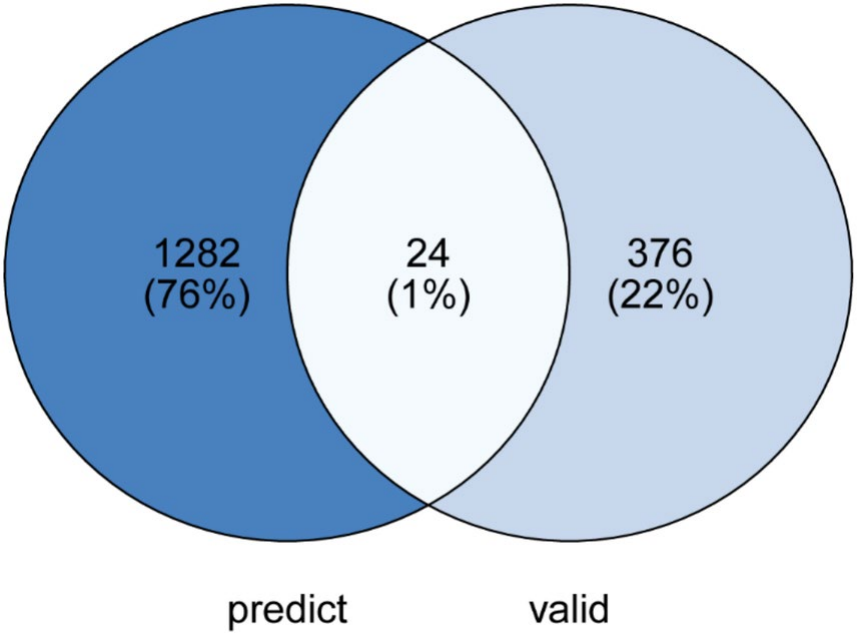
B vs. A_miRNA target overlap. The percentages and numbers reflect the distribution and overlap of miRNA targets between the two conditions.

Further analysis combined the results from the prediction model and experimental validation to filter miRNA target genes. By using Figure 10 (B vs. A miRNA target overlap analysis, Venn diagram) and Table 2 (miRNA target gene scores and experimental validation data), we deeply explored the relationship between prediction and experimental validation.

**Table 2:** Overlapping targets between B and A groups.

mature_mirna_id	target_symbol	Score	Experiment
rno-miR-125b-5p	Sertad3	0.714	Degradome sequencing
rno-miR-125b-5p	Lactb	0.714	Degradome sequencing
rno-miR-125b-5p	Sema4b	0.714	Degradome sequencing
rno-miR-125b-5p	Masp1	0.714	Degradome sequencing
rno-miR-125b-5p	Lrp4	0.714	Degradome sequencing
rno-miR-125b-5p	Tnfsf4	0.714	Degradome sequencing
rno-miR-125b-5p	Sertad3	18.6442	Degradome sequencing
rno-miR-125b-5p	Nmnat1	18.3062	Degradome sequencing
rno-miR-125b-5p	NA	18.0941	Degradome sequencing
rno-miR-125b-5p	NA	18.0829	Degradome sequencing
rno-miR-125b-5p	Sertad3	95.30908	Degradome sequencing
rno-miR-125b-5p	Tnfsf4	93.63888	Degradome sequencing
rno-miR-125b-5p	Lactb	92.66533	Degradome sequencing
rno-miR-125b-5p	Prdm1	91.68861	Degradome sequencing
rno-miR-125b-5p	G4	90.42021	Degradome sequencing
rno-miR-125b-5p	Cyp24a1	90.35977	Degradome sequencing

mature_mirna_id: The ID of the mature miRNA, representing the differentially expressed miRNAs in this study.

target_symbol: The symbol or name of the target gene, representing the gene targeted by each miRNA.

Score: The binding score between the target gene and miRNA, reflecting the potential regulatory effect of the miRNA on the target gene. A higher score indicates a stronger binding ability.

Experiment: The experimental method used; in this study, “Degradome sequencing” was used, which is a commonly used method for validating miRNA target genes.

This table shows the target genes of differentially expressed miRNAs between Groups B and A, validated using Degradome sequencing. Target genes with higher scores indicate stronger interactions with the miRNA. “NA” in the table indicates missing data, as some target genes may not have relevant data under certain miRNAs.

The Venn diagram in Figure 10 shows that 76% (1,282) of the miRNA target genes were identified as effective only in the prediction model, but not confirmed in the experimental validation. This indicates that although these target genes were predicted to be potentially effective, their expected effects were not verified experimentally, suggesting that the prediction model may have some error. However, this result also reflects the initial effectiveness of the prediction model in screening potential target genes.

On the other hand, 22% (376) of the target genes were confirmed to be effective only in the experimental validation but were not identified in the prediction model. This suggests that the prediction model missed some target genes, implying that the model needs to be optimized to improve its ability to recognize all relevant target genes. Notably, only 1% (24) of the target genes were shown to be effective in both the prediction and experimental validation, which, although a small proportion, highlights the potential of the current model in recognizing verified target genes.

According to the data in Table 2, although some target genes had high scores in the prediction model, their effectiveness was not confirmed in experimental validation, reflecting the limitations of the prediction model. At the same time, some target genes that were experimentally validated as effective, despite having low scores in the prediction model, showed significant biological effects, further confirming the potential of the prediction model in identifying new target genes.

## 4 Discussion

This study, through high-throughput miRNA sequencing technology, revealed the significant impact of hemorrhagic stress on the expression of plasma exosome miRNAs in rats and explored the potential roles of miRNAs in immune response, vascular repair, and cellular repair. Compared to existing literature, the study provides new evidence for the role of miRNAs in hemorrhagic stress and offers potential biomarkers and therapeutic targets for clinical intervention.

### 4.1 The impact of hemorrhagic stress on exosomal miRNA expression

Our study found that hemorrhagic shock significantly altered the expression profiles of multiple miRNAs in the plasma exosomes of rats. Specifically, miRNA-193b-3p was significantly upregulated in the hemorrhagic shock group, while miRNA-485-5p was significantly downregulated (42). The upregulation of miRNA-193b-3p suggests that it may play a positive role in vascular repair. Previous studies have shown that miRNA-193b regulates the vascular repair process by targeting angiogenesis-related genes such as VEGF (43). This finding further confirms the potential of miRNA-193b-3p in promoting vascular repair after hemorrhage. Additionally, the downregulation of miRNA-485-5p is closely associated with the impairment of cell repair mechanisms, suggesting that it may influence tissue recovery by regulating oxidative stress responses (44). Relevant literature indicates that miRNA-485-5p plays an important role in antioxidant responses and cell repair (45). Our study further supports this view, suggesting that the downregulation of miRNA-485-5p may impact the cell repair process after hemorrhage.

However, although the role of miRNA-21 in hemorrhagic shock has been reported, indicating its anti-inflammatory effect in immune responses and its promotion of cell repair (46), our study did not observe significant expression changes of miRNA-21 in hemorrhagic shock. This may be due to differences in experimental design or model systems. Therefore, future studies should further explore the role of miRNA-21 under different stress conditions and its expression patterns in plasma exosomes (47).

The biological significance of these altered miRNAs lies in their role as active ‘shuttles’ in exosome-mediated intercellular communication. During hemorrhagic shock, plasma exosome cargo reflects the physiological state of stressed parent cells, such as vascular endothelial and immune cells. Once released into circulation, these exosomes are selectively internalized by distant recipient cells, where miRNAs post-transcriptionally regulate target genes. For instance, the observed differential expression of miRNA-193b-3p and miRNA-485-5p—associated with VEGF, TNF, and MAPK pathways—likely represents a sophisticated messaging system between damaged tissues and the systemic environment to maintain homeostasis under stress.

### 4.2 The role of miRNA in immune response

The role of miRNAs in immune response is one of the key findings of this study. Research has shown that miRNAs, by regulating the function of immune cells and immune responses, may play a critical role in immune regulation after hemorrhagic shock (48). For example, miRNA-155 plays a key role in immune responses, especially in T cell activation and inflammation (49). In this study, we found that the upregulation of miRNA-193b-3p may contribute to the enhancement of immune responses after hemorrhagic shock, further validating the potential role of miRNAs in immune regulation. KEGG pathway analysis revealed that the target genes of differentially expressed miRNAs were significantly enriched in the TNF and MAPK signaling pathways, both of which are closely related to immune response and cell apoptosis (50). Further analysis showed that the downregulation of miRNA-1b may be associated with increased cell apoptosis and exacerbated tissue damage (51). This finding provides new insights into the cell repair process after hemorrhage. Previous studies have extensively researched the role of the miRNA-1 family in cell apoptosis and cardiovascular diseases, suggesting that miRNA-1b may play an important role in the coordinated mechanisms of immune and cell repair.

Although miRNA-155 and miRNA-193b-3p play significant roles in immune regulation, we should also pay attention to the other functions of miRNAs beyond immune cell activation. For example, miRNAs may influence immune responses by regulating cytokine release and immune cell differentiation (52). Therefore, future research should further explore the multiple roles of miRNAs in immune responses.

### 4.3 The potential role of miRNA in vascular repair

The role of miRNAs in vascular repair is one of the key findings of this study. We found that the upregulation of miRNA-193b-3p may be closely related to angiogenesis and the repair process. Vascular repair is crucial for restoring blood flow and tissue function after hemorrhage (53). miRNA-193b-3p may promote vascular repair after hemorrhage by targeting angiogenesis-related genes such as VEGF (54). However, Liu et al. proposed that, under certain inflammatory conditions, the upregulation of miRNA-193b-3p may limit angiogenesis. Therefore, future research should further explore the mechanism of miRNA-193b-3p under different physiological conditions, especially the impact of immune responses and the inflammatory environment after hemorrhagic shock on its function (55).

Additionally, our study also found that the role of miRNA-193b-3p in immune responses may be interconnected with vascular repair. By regulating the expression of relevant genes in immune responses, miRNA-193b-3p may promote vascular repair (56). This finding provides a new perspective for future studies on the multiple roles of miRNAs in angiogenesis.

### 4.4 The role of miRNA in cell repair and antioxidant responses

The role of miRNAs in cell repair and oxidative stress responses is another important aspect of hemorrhagic shock research. The downregulation of miRNA-485-5p may affect cell repair mechanisms, leading to delayed tissue repair (57). The role of miRNA-485-5p in oxidative stress and cell repair has been confirmed in several studies (58). Our research further supports this view, suggesting that the downregulation of miRNA-485-5p may be closely related to the impairment of the cell repair process after hemorrhage (59). Oxidative stress plays an important role in the cell repair process, so miRNA-485-5p may play a key role in regulating the oxidative stress response (60).

Compared to existing research, our findings indicate that the role of miRNA-485-5p may be closely related to changes in tissue and pathological conditions (61). Therefore, future research needs to delve deeper into the mechanisms of miRNA-485-5p under different pathological conditions, especially its regulatory role in oxidative stress and cell repair processes (62).

### 4.5 Limitations of the study and future directions

Although this study reveals the impact of hemorrhagic shock on exosome miRNAs, there are still some limitations. First, this study only used a rat model, which may affect the generalizability of the results. Although rats are commonly used in hemorrhagic shock research, whether the results can be fully applied to humans still requires further validation (63). Second, the changes in miRNA expression in this study were mainly analyzed using high-throughput sequencing technology. While this method has high sensitivity and accuracy, there may still be false positive or false negative results (64). Therefore, future research could use methods like qPCR to validate the differentially expressed miRNAs to improve the reliability of the results.

Furthermore, the specific mechanisms of miRNAs are not yet fully understood. Future studies should further explore the target genes and mechanisms of these miRNAs to uncover their specific functions in hemorrhagic shock (65). Future research should focus on multi-model validation, target gene verification of miRNAs, exploring the roles of miRNA-21 and other miRNAs, and deeply investigating the mechanisms of miRNAs in hemorrhagic shock. Additionally, the potential of miRNAs as biomarkers and therapeutic targets should be evaluated (66).

Regarding the characterization of the isolated vesicles, it should be noted that we verified the plasma exosomes primarily through Scanning Electron Microscopy (SEM) and Dynamic Light Scattering (DLS). The SEM images revealed typical cup-shaped vesicular structures with clear lipid bilayers, and the DLS analysis confirmed a consistent particle size distribution predominantly between 80 and 90 nm. Although the latest guidelines, such as MISEV2023, recommend the molecular validation of specific transmembrane protein markers like CD63, CD9, and CD81 for standardization, this study prioritized the yield and quality of exosomal RNA to ensure the accuracy of high-throughput sequencing, given the limited total volume of plasma samples available. Consequently, protein-level validation via Western Blot or flow cytometry was not performed in this current phase. In future research, we plan to include these molecular markers to further standardize exosome identification.

Another important limitation of this study is the lack of specificity validation of the identified miRNAs. While our results demonstrate that miRNA-193b-3p and miRNA-485-5p are significantly altered under hemorrhagic stress, their specificity as potential biomarkers and therapeutic targets remains to be confirmed. These miRNAs may also exhibit expression changes in other types of stress responses (e.g., ischemic stress, inflammatory stress) or cardiovascular diseases, which could limit their clinical application value.

In future research, we will systematically evaluate the specificity of these two miRNAs by comparing their expression levels in rat models of myocardial ischemia, sepsis, and traumatic brain injury. Furthermore, we will validate their diagnostic specificity in clinical cohorts by comparing plasma exosomal miRNA levels between patients with hemorrhagic shock and those with other acute critical illnesses, which will lay a solid foundation for their translation into clinical biomarkers and therapeutic agents.

Finally, a more detailed mechanistic correlation verified by other techniques should be included in future research. Specifically, luciferase reporter assays on specific promoters should be performed to confirm the direct binding and regulatory relationship between the identified miRNAs and their predicted target genes, providing more robust evidence of the molecular pathways involved. Moreover, the effect of “confounder” factors should be taken into account in future experimental designs. Factors such as gender and age can significantly influence physiological responses to blood loss and miRNA expression dynamics. Additionally, the time interval from hemorrhage induction to sampling, as well as the specific time of the day (circadian rhythm), are critical variables, as miRNA expression profiles are known to be highly time-dependent. Addressing these confounding factors through standardized protocols and longitudinal studies will be essential to validate the robustness and clinical relevance of exosomal miRNAs in the context of hemorrhagic stress.

## 5 Conclusion

This study, using high-throughput miRNA sequencing technology, explored the impact of hemorrhagic stress on miRNA expression in rat plasma exosomes. It revealed that hemorrhagic stress significantly altered the expression profiles of multiple miRNAs, particularly the upregulation of miRNA-193b-3p, which is associated with the vascular repair process, and the downregulation of miRNA-485-5p, which may be linked to impaired cell repair and antioxidant responses. Further target gene prediction and pathway analysis indicated that differentially expressed miRNAs play potential roles in key physiological processes such as immune response, cell repair, and angiogenesis, with significant enrichment in the TNF and MAPK signaling pathways. These findings suggest that these miRNAs may play important roles in the recovery process after blood loss by regulating these pathways. These results provide new evidence for the biological functions of miRNAs in hemorrhagic stress and offer potential biomarkers and therapeutic targets for early diagnosis and intervention. Additionally, this study found that changes in the expression of miRNAs such as miRNA-193b-3p and miRNA-485-5p may be closely related to alterations in immune regulation and cell repair processes, further validating the potential role of miRNAs in immune response and tissue repair.

Although this study provides new insights into the role of exosomal miRNAs in hemorrhagic stress, there are certain limitations, such as the use of only a rat model, and the broad applicability of the results needs further validation through other animal models or clinical studies. Overall, the findings suggest that exosomal miRNAs have significant potential in hemorrhagic stress, particularly in early diagnosis, immune regulation, and cell repair, with important clinical application prospects.

## Data Availability

The datasets presented in this study can be found in online repositories. The names of the repository/repositories and accession number(s) can be found at: PRJNA1417459 (Bioproject, NCBI).
