# Supplementary material for: Study on plasma exosome miRNA sequencing and analysis in rats under hemorrhagic stress
Source: Front Med (Lausanne). 2026 Apr 16;13:1798252. doi: 10.3389/fmed.2026.1798252 (PMC13128405; doi:10.3389/fmed.2026.1798252)
Supplement: Supplementary file 1 [file Table_1.DOCX]

Supplementary document

**Figure S1**: Morphology of exosomes from the DSXJ_A and DSXJ_B groups (with particle size ranges of 500 nm and 1 µm).


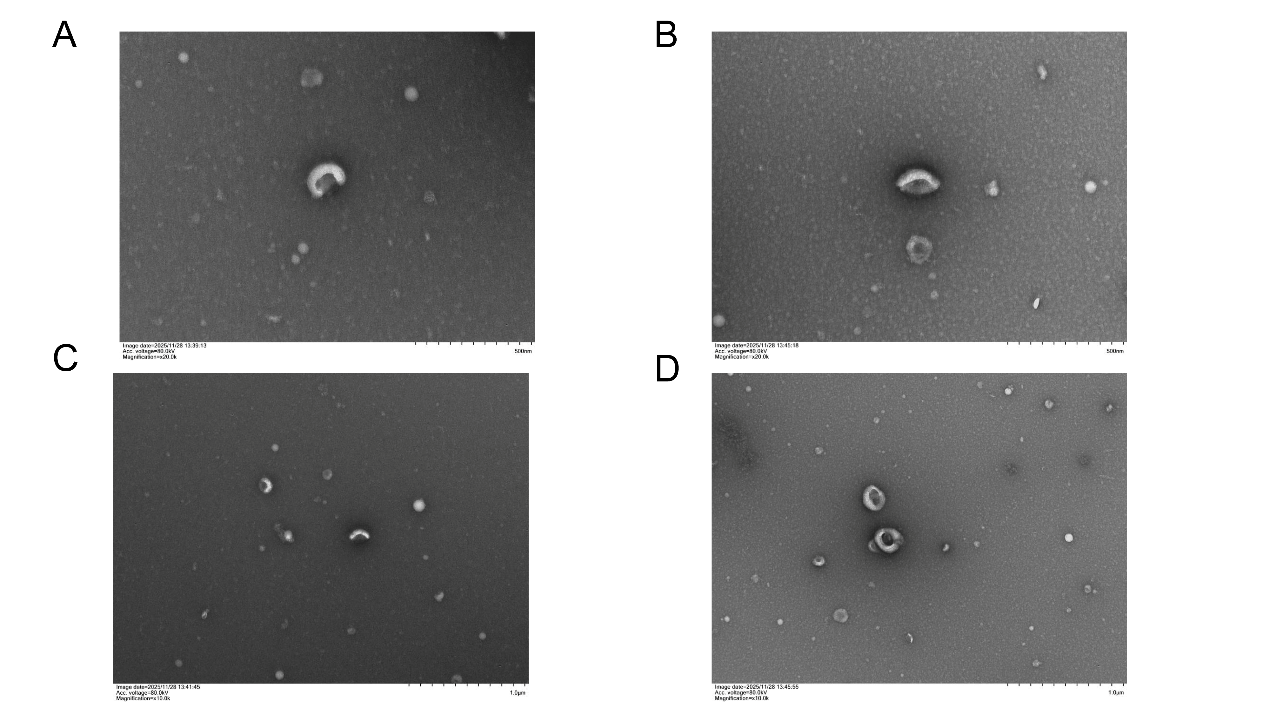


**Note**: Figure 2 shows the scanning electron microscope (SEM) images of exosomes from the DSXJ_A and DSXJ_B groups. Panels A and B display the morphology of exosomes from the DSXJ_A group (500 nm particle size range) and the DSXJ_B group (500 nm particle size range), respectively. Panels C and D show the morphology of exosomes from the DSXJ_A group (1 µm particle size range) and the DSXJ_B group (1 µm particle size range), respectively. All images exhibit a typical bilayer membrane structure, with particle size ranges primarily concentrated around 500 nm and 1 µm, respectively. The exosome morphology is relatively uniform, with no obvious impurities or particles observed.

**Table S1**: Small RNA length read data.

| seq_length | DSXJE_A1 | DSXJE_A2 | SXJE_A3 | DSXJE_B1 | DSXJE_B2 | DSXJE_B3 |
| --- | --- | --- | --- | --- | --- | --- |
| 17 | 1250788 | 1271738 | 726794 | 1134722 | 940935 | 869776 |
| 22 | 2889879 | 2711185 | 1857696 | 2764650 | 2106311 | 2044798 |
| 27 | 1872861 | 1620451 | 958099 | 1539499 | 1318163 | 1253973 |
| 32 | 1616631 | 1420468 | 917919 | 1068817 | 1087086 | 979560 |
| 37 | 962558 | 762623 | 553467 | 665134 | 684326 | 609265 |
| 42 | 400888 | 342786 | 274388 | 279033 | 276740 | 270395 |
| 47 | 242324 | 196985 | 179793 | 230711 | 181412 | 186734 |
| 52 | 130763 | 110553 | 122677 | 123560 | 101262 | 108192 |
| 57 | 94987 | 79871 | 112169 | 99545 | 71825 | 84203 |
| 62 | 62925 | 49515 | 72695 | 51434 | 41560 | 59519 |
| 67 | 32027 | 25409 | 39554 | 28341 | 20781 | 25471 |
| 72 | 20237 | 14536 | 27170 | 19088 | 13515 | 17343 |
| 77 | 12055 | 7183 | 14330 | 11373 | 8207 | 8874 |
| 82 | 7810 | 6572 | 10313 | 7971 | 5512 | 5990 |
| 87 | 6052 | 3715 | 7977 | 6676 | 4105 | 4768 |
| 92 | 4342 | 2277 | 5385 | 3948 | 3048 | 3210 |
| 97 | 3236 | 1640 | 4388 | 3014 | 2488 | 2392 |
| 102 | 3359 | 1550 | 4056 | 2679 | 2510 | 2393 |
| 107 | 2767 | 1389 | 4210 | 2582 | 2115 | 2050 |
| 112 | 12043 | 6454 | 15147 | 6063 | 5042 | 7140 |
| 117 | 10743 | 3085 | 4448 | 4495 | 5570 | 4758 |
| 122 | 7109 | 2962 | 6395 | 6354 | 6369 | 4348 |
| 127 | 6900 | 3833 | 8911 | 4976 | 2794 | 5393 |
| 132 | 1026 | 624 | 1101 | 1213 | 925 | 791 |
| 137 | 563 | 372 | 721 | 639 | 419 | 503 |
| 142 | 585 | 375 | 756 | 908 | 487 | 552 |
| 147 | 44 | 23 | 37 | 59 | 31 | 19 |
| 150 | 5631 | 4134 | 10746 | 17434 | 5121 | 6319 |

Note: The data shows the distribution of different sequence lengths (seq_length) in each sample. The seq_length in the table represents the length of the small RNA, measured in base pairs (bp). Each sample column shows the corresponding number of small RNA reads. These data help in further analyzing the expression differences of small RNAs of different lengths in various samples and their biological significance.

**Table S2**: Sequencing and quality information of the data.

| SampleID | reads_raw | Q20_raw | Q30_raw | raw_GC% | reads_clean | Q20_clean | Q30_clean | clean_GC% |
| --- | --- | --- | --- | --- | --- | --- | --- | --- |
| DSXJE_A1_R1 | 14036774 | 91.21% | 84.57% | 59% | 13893238 | 98.78% | 96.10% | 52% |
| DSXJE_A1_R2 | 14036774 | 94.04% | 88.85% | 55% | 13893238 | 98.89% | 96.32% | 52% |
| DSXJE_A2_R1 | 14177674 | 94.56% | 89.14% | 59% | 14048968 | 98.77% | 96.02% | 52% |
| DSXJE_A2_R2 | 14177674 | 94.10% | 88.86% | 54% | 14048968 | 99.16% | 96.98% | 52% |
| DSXJE_A3_R1 | 9688469 | 95.08% | 89.67% | 57% | 9581225 | 98.72% | 95.98% | 51% |
| DSXJE_A3_R2 | 9688469 | 95.65% | 90.42% | 55% | 9581225 | 98.41% | 95.14% | 51% |
| DSXJE_B1_R1 | 14587702 | 94.04% | 88.37% | 58% | 14451382 | 98.47% | 95.46% | 51% |
| DSXJE_B1_R2 | 14587702 | 96.25% | 92.11% | 56% | 14451382 | 99.20% | 97.25% | 51% |
| DSXJE_B2_R1 | 10179625 | 93.03% | 86.31% | 57% | 10083680 | 98.61% | 95.66% | 52% |
| DSXJE_B2_R2 | 10179625 | 96.38% | 91.95% | 56% | 10083680 | 98.94% | 96.53% | 52% |
| DSXJE_B3_R1 | 10356636 | 95.21% | 90.00% | 57% | 10243726 | 98.66% | 95.82% | 51% |
| DSXJE_B3_R2 | 10356636 | 95.92% | 90.88% | 54% | 10243726 | 98.65% | 95.63% | 51% |

Note: **Table S2** displays the sequencing data quality for each sample, including raw data and cleaned data metrics. The table lists the sequencing depth, the proportion of Q20 and Q30, GC content, and other quality control parameters for each sample. reads_raw represents the number of raw reads, while Q20_r and Q30_r indicate the proportion of Q20 and Q30 in the raw data, respectively. raw_GC% refers to the GC content of the raw data. reads_clean shows the number of cleaned reads, with Q20_cl and Q30_cl representing the proportion of Q20 and Q30 in the cleaned data, respectively. clean_GC% refers to the GC content in the cleaned data. In the data, Q20 and Q30 represent the proportions of sequencing quality scores (quality scores 20 and 30), with higher Q-values indicating better sequencing quality. GC content refers to the proportion of guanine (G) and cytosine (C) base pairs in the sample, and this parameter helps assess the composition of the sequencing data and its potential biological characteristics.

**Table S3**: Rfam database alignment statistical analysis.

| sampleID | Cis-reg | lncRNA | other sRNA | others | rRNA | snoRNA | total |
| --- | --- | --- | --- | --- | --- | --- | --- |
| DSXJE_A1 | 9756 | 725 | 6661 | 918330 | 145034 | 515 | 1081021 |
| DSXJE_A2 | 9844 | 825 | 7507 | 716924 | 120597 | 544 | 856241 |
| DSXJE_A3 | 6281 | 380 | 4206 | 462098 | 99694 | 296 | 572955 |
| DSXJE_B1 | 9618 | 552 | 6623 | 738586 | 145808 | 538 | 901725 |
| DSXJE_B2 | 7482 | 488 | 4967 | 632847 | 107359 | 348 | 753491 |
| DSXJE_B3 | 8100 | 429 | 5490 | 573241 | 109025 | 297 | 696582 |

**Table S 3**: Rfam Database Alignment Statistical Analysis.

Table 2 shows the alignment statistics of each sample in the Rfam database. The table lists the alignment numbers of each sample in different small RNA categories, including Cis-reg, lncRNA, other sRNA, others, rRNA, snoRNA, and calculates the alignment numbers for each category and the total. The specific meaning of each column is as follows:

- **Cis-reg**: The number of alignments related to cis-regulatory RNA.
- **lncRNA**: The number of alignments for long non-coding RNA.
- **other sRNA**: The number of alignments for other types of small RNA.
- **others**: The number of alignments for other uncategorized small RNA.
- **rRNA**: The number of alignments for ribosomal RNA.
- **snoRNA**: The number of alignments for small nucleolar RNA.
- **total**: The total number of alignments for each sample.

**Note**: The data in this table comes from the alignment results of the Rfam database, covering the alignment of various small RNA types. The alignment results for each sample help analyze the distribution and enrichment of different types of small RNA, providing a basis for further functional studies and expression analysis. The **total** column shows the total alignment count of all small RNAs in all categories for each sample, reflecting the comprehensiveness and overall quality of the data.

**Table S 4**: miRNA expression analysis after filtering.

| miRNA | total | DSXJE_A1.norm | DSXJE_A2.norm | DSXJE_A3.norm | DSXJE_B1.norm | DSXJE_B2.norm | DSXJE_B3.norm |
| --- | --- | --- | --- | --- | --- | --- | --- |
| rno-let-7a-5p | 308347.7 | 109756.7 | 109117.9 | 109811.5 | 108556.2 | 102814.2 | 113211.96 |
| rno-miR-191a-5p | 313355.9 | 122958.9 | 92907.78 | 99308.86 | 98959.35 | 120981.1 | 126022.09 |
| rno-let-7f-5p | 228864.7 | 79884.74 | 79805.09 | 81078.46 | 82225.58 | 77596.53 | 85623.94 |
| rno-let-7c-5p | 211937.9 | 73635.92 | 73924.8 | 69949.86 | 81895.77 | 72723.42 | 68971.84 |
| rno-let-7i-5p | 145730.3 | 53454.28 | 55614.89 | 53358.59 | 47509.46 | 49616.56 | 52931.29 |
| rno-let-7b-5p | 125656.6 | 44201.58 | 47783.91 | 42720.43 | 46441.9 | 41457.59 | 41221.95 |
| rno-let-7g-5p | 116258.1 | 42566.13 | 44458.86 | 39893.91 | 39684.42 | 39797.95 | 40092.38 |
| rno-miR-26a-5p | 107688.7 | 36350.69 | 38324.66 | 39229.23 | 40042.75 | 39513.91 | 36984.28 |
| rno-miR-16-5p | 79138.65 | 26122.65 | 33105.35 | 34860.38 | 26649.29 | 30313.49 | 25404.43 |
| rno-let-7d-5p | 66695.78 | 24301.78 | 21377.01 | 24380.33 | 23315.19 | 22722.59 | 23922.33 |

**Note**:

- The **total** column displays the total expression level of each miRNA, without any treatment applied.
- The other columns show the expression data of each miRNA in different experimental groups (Group A, Group B). These data have been normalized to allow for more accurate comparisons of expression levels between different groups and samples.
- **Filtering Criteria**: The data were filtered based on the condition that the expression level is greater than 200 in 90% of the samples. Only miRNAs that meet this filtering criterion are retained, ensuring that the selected miRNAs have significant expression in most samples.

**Table S 5**: Differential miRNA analysis between B and A groups after filtering.

| miRNA | Alias | Length | baseMean | log2FoldChange | pvalue | padj |
| --- | --- | --- | --- | --- | --- | --- |
| rno-miR-193b-3p | MIMAT0035734 | 19 | 2.120014 | 4.378603 | 0.063648 | 0.987408 |
| rno-miR-485-5p | MIMAT0003203 | 22 | 1.535772 | -4.22302 | 0.097064 | 0.987408 |
| rno-miR-92b-3p | MIMAT0005340 | 22 | 1.361429 | -4.0495 | 0.129804 | 0.987408 |
| rno-miR-205 | MIMAT0000878 | 23 | 1.54724 | 3.919593 | 0.129405 | 0.987408 |
| rno-miR-1b | MIMAT0037263 | 22 | 12.86236 | 3.75755 | 5.54E-05 | 0.016617 |
| rno-miR-346 | MIMAT0000596 | 23 | 1.017502 | -3.62866 | 0.267707 | 0.987408 |
| rno-miR-674-3p | MIMAT0005330 | 22 | 1.187722 | 3.602489 | 0.19636 | 0.987408 |
| rno-miR-1843a-3p | MIMAT0024848 | 22 | 0.850298 | -3.37079 | 0.291129 | 0.987408 |
| rno-miR-204-3p | MIMAT0004739 | 22 | 0.847919 | -3.36578 | 0.336908 | 0.987408 |
| rno-miR-802-3p | MIMAT0017362 | 23 | 0.847919 | -3.36578 | 0.336908 | 0.987408 |

Note: This table shows the top 10 differentially expressed miRNAs between Group B and Group A, with a filtering criterion of an absolute log2FoldChange greater than 1.5, and the results are sorted in descending order based on the absolute value of log2FoldChange. The data in the table provide important candidate miRNAs for subsequent biological analysis. Each row displays the relevant information for one miRNA, including:

- miRNA: The name of the miRNA.
- Alias: The alias of the miRNA.
- Length: The length of the miRNA, in base pairs (bp).
- baseMean: The average expression level of the miRNA across all samples.
- log2FoldChange: The log-transformed fold change (Log2 Fold Change) of the miRNA, indicating the expression change between Group B and Group A.
- pvalue: The raw P-value for the differential expression of the miRNA.
- padj: The adjusted P-value after multiple comparison correction.

**Table S 6**: GO analysis of verified target genes of differentially expressed miRNAs between B and A groups.

| ONTOLOGY | ID | Description | GeneRatio | RichFactor | zScore | pvalue | p.adjust | query | geneID |  |
| --- | --- | --- | --- | --- | --- | --- | --- | --- | --- | --- |
| BP | GO:0042044 | fluid transport | 0.02 | 0.17 | 6.69 | 0.00 | 0.05 | 0.04 | Aqp4/Slc4a11/Slc26a6/Edn1/Has2/Hyal2 | |
| BP | GO:0031663 | lipopolysaccharide-mediated signaling pathway | 0.02 | 0.13 | 6.16 | 0.00 | 0.05 | 0.04 | Irf3/Irak2/Nfkbia/Prdm1/Scarb1/Cx3cl1/Nfkbil1 | |
| BP | GO:0060317 | cardiac epithelial to mesenchymal transition | 0.02 | 0.16 | 6.47 | 0.00 | 0.05 | 0.04 | Acvr1/Spry1/Has2/Snai1/Tbx3/Bmp2 | |
| BP | GO:0042481 | regulation of odontogenesis | 0.01 | 0.21 | 6.99 | 0.00 | 0.05 | 0.04 | Dmrt3/Rspo2/Edn1/Csf1/Bmp2 | |
| BP | GO:0042487 | regulation of odontogenesis of dentin-containing tooth | 0.01 | 0.27 | 7.22 | 0.00 | 0.07 | 0.06 | Dmrt3/Rspo2/Csf1/Bmp2 | |
| BP | GO:0035809 | regulation of urine volume | 0.01 | 0.17 | 6.24 | 0.00 | 0.07 | 0.06 | Adrb2/Edn1/Adm/Has2/Hyal2 | |
| BP | GO:0060707 | trophoblast giant cell differentiation | 0.01 | 0.22 | 6.50 | 0.00 | 0.07 | 0.06 | Prdm1/Snai1/Nr2f2/Sox15 | |
| BP | GO:0045906 | negative regulation of vasoconstriction | 0.01 | 0.21 | 6.29 | 0.00 | 0.07 | 0.06 | Bmpr2/Adm/Apln/Cx3cl1 | |
| MF | GO:0033549 | MAP kinase phosphatase activity | 0.01 | 0.22 | 6.53 | 0.00 | 0.08 | 0.07 | Styxl2/Dusp1/Dusp6/Dusp4 | |
| MF | GO:0008330 | protein tyrosine/threonine phosphatase activity | 0.01 | 0.30 | 6.71 | 0.00 | 0.09 | 0.09 | Dusp1/Dusp6/Dusp4 | |
| BP | GO:0042482 | positive regulation of odontogenesis | 0.01 | 0.30 | 6.69 | 0.00 | 0.11 | 0.09 | Edn1/Csf1/Bmp2 | |
| BP | GO:0006833 | water transport | 0.01 | 0.16 | 5.32 | 0.00 | 0.11 | 0.09 | Aqp4/Slc4a11/Has2/Hyal2 | |
| BP | GO:0003097 | renal water transport | 0.01 | 0.25 | 6.03 | 0.00 | 0.11 | 0.09 | Aqp4/Has2/Hyal2 | |
| BP | GO:0016338 | calcium-independent cell-cell adhesion via plasma membrane cell-adhesion molecules | 0.01 | 0.25 | 6.03 | 0.00 | 0.11 | 0.09 | Cldn11/Cx3cl1/Bmp2 | |
| BP | GO:0036302 | atrioventricular canal development | 0.01 | 0.25 | 6.03 | 0.00 | 0.11 | 0.09 | Has2/Tbx3/Bmp2 | |
| BP | GO:0038203 | TORC2 signaling | 0.01 | 0.25 | 6.03 | 0.00 | 0.11 | 0.09 | Prr5/Prr5l/Nckap1l | |
| BP | GO:0043652 | engulfment of apoptotic cell | 0.01 | 0.23 | 5.75 | 0.00 | 0.11 | 0.09 | Xkr8/Xkr9/Alox15 | |
| BP | GO:0035810 | positive regulation of urine volume | 0.01 | 0.20 | 5.29 | 0.00 | 0.11 | 0.10 | Edn1/Has2/Hyal2 | |
| MF | GO:0017017 | MAP kinase tyrosine/serine/threonine phosphatase activity | 0.01 | 0.23 | 5.78 | 0.00 | 0.13 | 0.12 | Dusp1/Dusp6/Dusp4 | |
| MF | GO:0045236 | CXCR chemokine receptor binding | 0.01 | 0.21 | 5.53 | 0.00 | 0.13 | 0.13 | Cxcl1/Cxcl2/Cx3cl1 | |
| CC | GO:0038201 | TOR complex | 0.01 | 0.25 | 5.99 | 0.00 | 0.39 | 0.39 | Prr5/Prr5l/Mlst8 | |

- ONTOLOGY: The type of GO term, with the main focus on Biological Process (BP) in this table.
- ID: The unique identifier for each GO term, following the standard GO system.
- Description: A brief description of the biological process associated with the GO term.
- GeneRatio: The ratio of target genes involved in a specific GO term to the total number of genes in the queried gene set.
- RichFactor: The enrichment factor, representing the ratio of target genes involved in a specific GO term to the total number of genes in that GO term.
- zScore: The standardized score, representing the strength of the association between the target genes and the GO term.
- pvalue: The raw P-value representing the statistical significance of the GO term in the target gene set.
- p.adjust: The adjusted P-value after methods such as Benjamini-Hochberg correction, accounting for multiple comparisons.
- query: The P-value threshold used to select significant GO terms.
- geneID: The list of target genes associated with each GO term.

Filtering Criteria:
To ensure statistical significance and highlight the most relevant biological processes, the following filtering criteria were applied:

1. p-value < 0.05: Only GO terms with a p-value less than 0.05 are included to ensure statistical significance.
2. RichFactor > 5: GO terms with a RichFactor greater than 5 are selected, emphasizing the significant enrichment of target genes in specific biological processes.
3. z-score > 5: GO terms with a z-score greater than 5 are filtered to further ensure a high level of significance in the gene set.

**Table S 7**: KEGG pathway analysis of verified target genes of differentially expressed miRNAs between B and A groups.

| subcategory | ID | Description | GeneRatio | BgRatio | RichFactor | zScore | query | p.adjust | qvalue |
| --- | --- | --- | --- | --- | --- | --- | --- | --- | --- |
| Signal transduction | rno04668 | TNF signaling pathway | 0.07 | 119/11099 | 0.10 | 7.49 | 0.00 | 0.00 | 0.00 |
| Signal transduction | rno04064 | NF-kappa B signaling pathway | 0.05 | 100/11099 | 0.08 | 5.18 | 0.00 | 0.02 | 0.02 |
| Transport and catabolism | rno04148 | Efferocytosis | 0.06 | 162/11099 | 0.06 | 4.73 | 0.00 | 0.02 | 0.02 |
| Signal transduction | rno04010 | MAPK signaling pathway | 0.08 | 302/11099 | 0.05 | 4.33 | 0.00 | 0.02 | 0.02 |
| Cardiovascular disease | rno05417 | Lipid and atherosclerosis | 0.06 | 223/11099 | 0.05 | 4.06 | 0.00 | 0.04 | 0.04 |
| Cancer: specific types | rno05218 | Melanoma | 0.03 | 76/11099 | 0.08 | 4.44 | 0.00 | 0.05 | 0.05 |
| Signal transduction | rno04350 | TGF-beta signaling pathway | 0.04 | 113/11099 | 0.06 | 3.96 | 0.00 | 0.07 | 0.06 |
| Signaling molecules and interaction | rno04061 | Viral protein interaction with cytokine and cytokine receptor | 0.03 | 84/11099 | 0.07 | 4.11 | 0.00 | 0.07 | 0.06 |
| Development and regeneration | rno04360 | Axon guidance | 0.05 | 184/11099 | 0.05 | 3.64 | 0.00 | 0.07 | 0.06 |
| Signaling molecules and interaction | rno04060 | Cytokine-cytokine receptor interaction | 0.06 | 274/11099 | 0.04 | 3.28 | 0.00 | 0.11 | 0.09 |
| Cell motility | rno04810 | Regulation of actin cytoskeleton | 0.06 | 240/11099 | 0.04 | 3.26 | 0.00 | 0.11 | 0.10 |
| Infectious disease: viral | rno05160 | Hepatitis C | 0.05 | 172/11099 | 0.05 | 3.26 | 0.01 | 0.12 | 0.10 |
| Infectious disease: parasitic | rno05140 | Leishmaniasis | 0.03 | 74/11099 | 0.07 | 3.59 | 0.01 | 0.12 | 0.10 |
| Infectious disease: bacterial | rno05135 | Yersinia infection | 0.04 | 139/11099 | 0.05 | 3.29 | 0.01 | 0.12 | 0.10 |
| Cancer: specific types | rno05215 | Prostate cancer | 0.03 | 108/11099 | 0.06 | 3.34 | 0.01 | 0.12 | 0.10 |
| Signal transduction | rno04015 | Rap1 signaling pathway | 0.05 | 217/11099 | 0.04 | 3.07 | 0.01 | 0.12 | 0.10 |
| Cancer: specific types | rno05214 | Glioma | 0.03 | 78/11099 | 0.06 | 3.44 | 0.01 | 0.12 | 0.10 |
| Immune system | rno04062 | Chemokine signaling pathway | 0.05 | 182/11099 | 0.04 | 3.08 | 0.01 | 0.12 | 0.10 |
| Nucleotide metabolism | rno00240 | Pyrimidine metabolism | 0.02 | 55/11099 | 0.07 | 3.40 | 0.01 | 0.15 | 0.13 |
| Cardiovascular disease | rno05418 | Fluid shear stress and atherosclerosis | 0.04 | 155/11099 | 0.05 | 2.96 | 0.01 | 0.15 | 0.13 |

- **Note**:
  - **subcategory**: The subcategory of the pathway, which describes which biological process or functional classification the pathway belongs to, such as signal transduction, transport, metabolism, etc.
  - **ID**: The unique identifier for each KEGG pathway, following KEGG database standards.
  - **Description**: The description related to the KEGG pathway, explaining the biological function of the pathway.
  - **GeneRatio**: The ratio of target genes in a specific KEGG pathway, representing the number of target genes in that pathway relative to the total number of genes in the queried gene set.
  - **BgRatio**: The background gene ratio, representing the ratio of the total number of genes in the KEGG pathway to the total number of genes in the entire genome.
  - **RichFactor**: The enrichment factor, indicating the degree of enrichment of target genes in the pathway. A larger enrichment factor indicates greater significant enrichment of target genes in the pathway.
  - **zScore**: The standardized z-score used to evaluate the correlation between target genes and the pathway. A higher z-score indicates a more significant relationship between the pathway and the target genes.
  - **query**: The p-value threshold used to select significant pathways.
  - **p.adjust**: The adjusted p-value after methods such as Benjamini-Hochberg correction, accounting for multiple hypothesis testing. A smaller adjusted p-value indicates a higher statistical significance of the pathway.
  - **qvalue**: The q-value provided, indicating the significance level after further adjustment of the p-value.

**Filtering Criteria**:
To ensure the statistical significance of the pathway analysis results, the following filtering criteria were applied in this study:

1. **p.adjust < 0.3**: An adjusted p-value less than 0.3 indicates that the pathway has some statistical significance.
2. **qvalue < 0.3**: A q-value less than 0.3 further ensures that the selected pathways have high reliability.
3. **RichFactor > 0.03**: Pathways with an enrichment factor greater than 0.03 are selected, emphasizing the high degree of enrichment of target genes in those pathways.
4. **GeneRatio > 0.02**: Pathways with a gene ratio greater than 0.02 are selected, indicating a higher proportion of target genes in that pathway.
